# Activation of EphA2-EGFR signaling in oral epithelial cells by *Candida albicans* virulence factors

**DOI:** 10.1371/journal.ppat.1009221

**Published:** 2021-01-20

**Authors:** Marc Swidergall, Norma V. Solis, Nicolas Millet, Manning Y. Huang, Jianfeng Lin, Quynh T. Phan, Michael D. Lazarus, Zeping Wang, Michael R. Yeaman, Aaron P. Mitchell, Scott G. Filler

**Affiliations:** 1 Division of Infectious Diseases, Harbor-UCLA Medical Center, Torrance, California, United States of America; 2 Institute for Infection and Immunity, The Lundquist Institute for Biomedical Innovation at Harbor-UCLA Medical Center, Torrance, California, United States of America; 3 David Geffen School of Medicine at UCLA, Los Angeles, California, United States of America; 4 Department of Biological Sciences, Carnegie Mellon University, Pittsburgh, Pennsylvania, United States of America; 5 Division of Molecular Medicine, Harbor-UCLA Medical Center, Torrance, California, United States of America; 6 Department of Microbiology, University of Georgia, Athens, Georgia, United States of America; University of Wisconsin-Madison, UNITED STATES

## Abstract

During oropharyngeal candidiasis (OPC), *Candida albicans* invades and damages oral epithelial cells, which respond by producing proinflammatory mediators that recruit phagocytes to foci of infection. The ephrin type-A receptor 2 (EphA2) detects β-glucan and plays a central role in stimulating epithelial cells to release proinflammatory mediators during OPC. The epidermal growth factor receptor (EGFR) also interacts with *C*. *albicans* and is known to be activated by the Als3 adhesin/invasin and the candidalysin pore-forming toxin. Here, we investigated the interactions among EphA2, EGFR, Als3 and candidalysin during OPC. We found that EGFR and EphA2 constitutively associate with each other as part of a heteromeric physical complex and are mutually dependent for *C*. *albicans-*induced activation. Als3-mediated endocytosis of a *C*. *albicans* hypha leads to the formation of an endocytic vacuole where candidalysin accumulates at high concentration. Thus, Als3 potentiates targeting of candidalysin, and both Als3 and candidalysin are required for *C*. *albicans* to cause maximal damage to oral epithelial cells, sustain activation of EphA2 and EGFR, and stimulate pro-inflammatory cytokine and chemokine secretion. In the mouse model of OPC, *C*. *albicans-*induced production of CXCL1/KC and CCL20 is dependent on the presence of candidalysin and EGFR, but independent of Als3. The production of IL-1α and IL-17A also requires candidalysin but is independent of Als3 and EGFR. The production of TNFα requires Als1, Als3, and candidalysin. Collectively, these results delineate the complex interplay among host cell receptors EphA2 and EGFR and *C*. *albicans* virulence factors Als1, Als3 and candidalysin during the induction of OPC and the resulting oral inflammatory response.

## Author summary

Oropharyngeal candidiasis occurs when the fungus *Candida albicans* proliferates in the mouth to a point at which tissue damage occurs. The disease is characterized by fungal invasion of the superficial epithelium and a localized inflammatory response. Two *C*. *albicans* virulence factors contribute to the pathogenesis of OPC, Als3 which enables the organism to adhere to and invade host cells, and candidalysin which is a pore-forming toxin that damages host cells. Two epithelial cell receptors, ephrin type-A receptor 2 (EphA2) and the epidermal growth factor receptor (EGFR) are activated by *C*. *albicans*. Here, we show that EphA2 and EGFR form part of complex wherein these co-receptors are required to activate each other. Als3 enhances the host cell targeting of candidalysin by stimulating epithelial cell endocytosis of *C*. *albicans*, leading to the formation of an endocytic vacuole in which candidalysin accumulates. Thus, Als3 and candidalysin synergize to damage epithelial cells, activate EphA2 and EGFR, and stimulate the production of inflammatory mediators. In the mouse model of OPC, candidalysin elicits of a subset of the oral inflammatory response molecular repertoire. Of the cytokines and chemokines induced by this toxin, some require the activation of EGFR while others are induced independently of EGFR. Collectively, this work provides a deeper understanding of the interactions among *C*. *albicans* virulence factors, host cell receptors and immune responses during OPC.

## Introduction

Oropharyngeal candidiasis is characterized by superficial fungal invasion of the oral mucosa, epithelial cell death, and leukocyte recruitment to the focus of infection [1]. *C*. *albicans* can invade the epithelial cell lining of the oropharynx by two mechanisms, active penetration and induced endocytosis. Active penetration occurs when a progressively elongating hypha physically pushes its way into the epithelial cell [2]. Induced endocytosis occurs when invasins such as Als1, Als3 and Ssa1 expressed by a hypha interact with epithelial cell receptors such as E-cadherin, HER2, and the epidermal growth factor receptor (EGFR), stimulating the epithelial cell to endocytose the organism [3–5]. *C*. *albicans* also damages epithelial cells by secreting candidalysin, a pore-forming toxin generated by the processing of Ece1 by the Kex1/2 proteases [6,7].

The epithelial cells that line the oropharynx sense the presence of *C*. *albicans* and orchestrate the host inflammatory response to fungal overgrowth. In addition to producing host defense peptides that have direct antifungal activity, oral epithelial cells secrete alarmins, proinflammatory cytokines, and chemokines that recruit phagocytes to foci of infection and enhance their candidacidal activity to limit the growth of the invading fungus [8–10]. This epithelial cell response is amplified by interleukin (IL)-17, which is secreted by γδ T cells, innate TCRαβ^+^ cells, and type-3 innate lymphoid cells [11–13].

Recently, it has become clear that the proinflammatory response to *C*. *albicans* is triggered when the fungus activates specific epithelial cell receptors. We determined that the ephrin type-A receptor 2 (EphA2) is an epithelial cell receptor tyrosine kinase that senses exposed β-glucan on the fungal surface. *C*. *albicans* yeast and hyphae interact with EphA2 on oral epithelial cells, stimulating EphA2 autophosphorylation and inducing the epithelial cells to secrete host defense peptides and proinflammatory mediators. In mice, EphA2 activation is required for the early production of IL-17A, and *Epha2*^–/–^mice are highly susceptible to the initial stages of OPC [10].

Another proinflammatory epithelial cell stimulus is candidalysin. This toxin causes an influx of calcium into the epithelial cells, which activates matrix metalloproteases and induces shedding of native EGFR ligands, leading to activation of EGFR signaling and a proinflammatory response by epithelial cells [14].

In the current study, we sought to elucidate the interactions among Als3, candidalysin, EphA2, and EGFR during *C*. *albicans* infection of oral epithelial cells. We found that when Als3-expressing hyphae are endocytosed by epithelial cells, candidalysin accumulates around the internalized portion of the organisms, resulting in a high local concentration of the toxin. *In vitro*, *C*. *albicans* cells must express both Als3 and Ece1 to activate epithelial cell EGFR, which maintains EphA2 phosphorylation and stimulates the secretion of CXCL8/IL-8 and CCL20. In mice with acute OPC, pharmacological inhibition of EGFR leads to a reduction in oral fungal burden and impaired inflammatory response during disease progression. Maximal mucosal infection requires either Als1 and Als3 or Ece1 while the mucosal inflammatory response is largely dependent on Ece1. Thus, *C*. *albicans* activation of EGFR mediates fungal invasion of the epithelium, which enhances the targeting of candidalysin and induces the local inflammatory responses to this fungus.

## Results

### EphA2 and EGFR functionally interact and physically associate with one another

**Fig 1:**
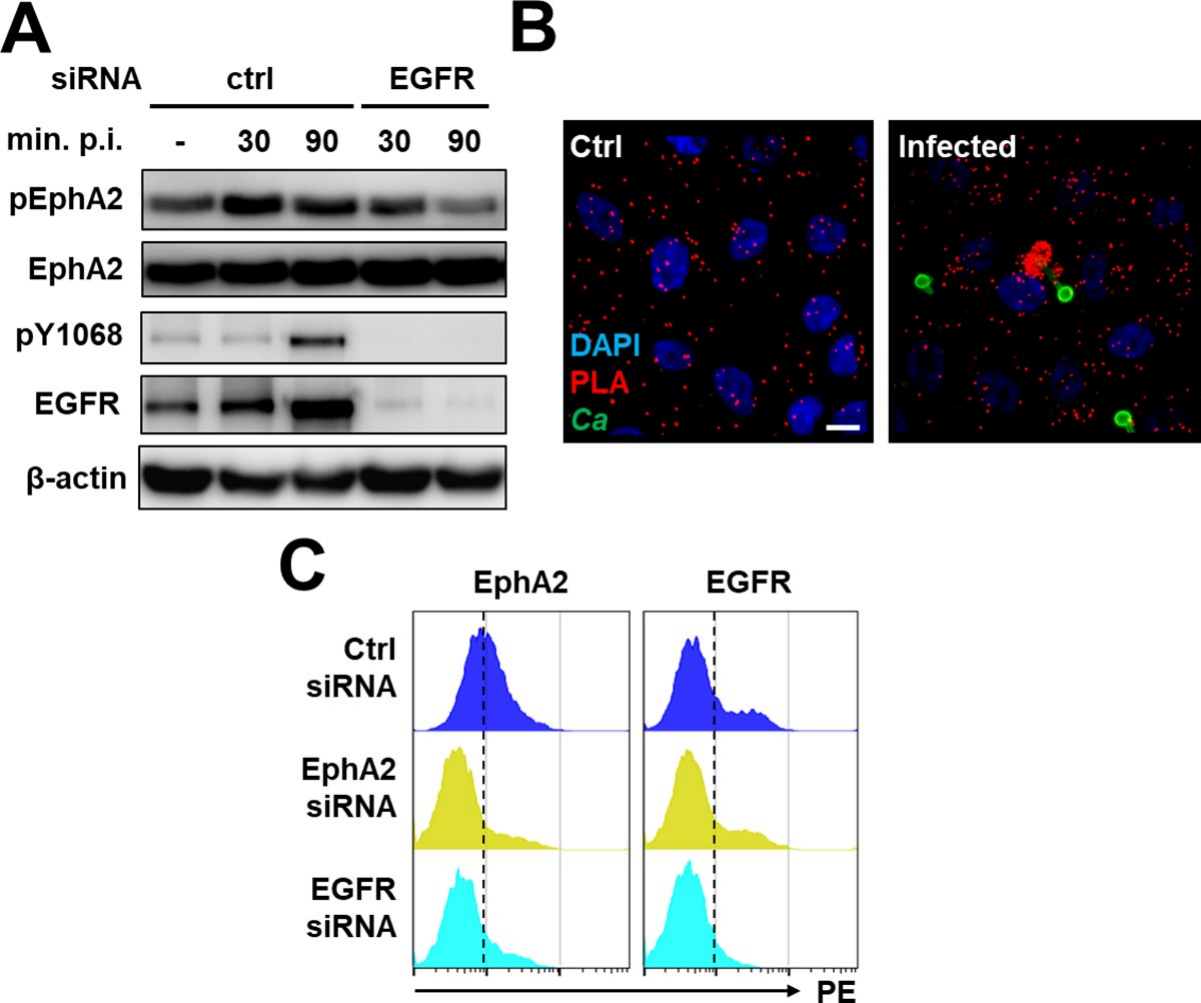
EphA2 and EGFR interact both functionally and physically. (A) Effects of epidermal growth factor (EGFR) siRNA or control (ctrl) siRNA on the time course of EphA2 and EGFR phosphorylation in OKF6/TERT-2 oral epithelial cells infected with *C*. *albicans*. Results are representative of 3 independent experiments. Densitometric quantification of all 3 immunoblots such as the one in (A) is shown in S1A Fig. (B) Proximity ligation assay showing the physical association between EphA2 and EGFR in uninfected (Ctrl) and *C*. *albicans* (Ca) infected epithelial cells after 90 min. Red spots indicate regions of EphA2-EGFR association. Scale bar 10 μm. (C) Effects of EphA2 and EGFR siRNA on the surface expression of EphA2 and EGFR by oral epithelial cells, as determined by flow cytometry. Immunoblots demonstrating the extent of siRNA knockdown in whole cell lysates are shown in S3A Fig. Results are representative of 3 independent experiments.

Previously, we found that infection of oral epithelial cells with viable *C*. *albicans* induces sustained phosphorylation of both EphA2 and EGFR, leading to the secretion of proinflammatory mediators [10]. By contrast, exposure of oral epithelial cells to purified β-glucan induces only transient EphA2 phosphorylation, does not activate EGFR, and is insufficient to induce a significant inflammatory response [10,15]. These results suggest that stimulation of a proinflammatory response in epithelial cells requires the sustained activation of EphA2 and/or activation of a second epithelial cell receptor such as EGFR. In our earlier work, we determined that the EphA2 must be activated for *C*. *albicans* to activate EGFR [10]. Here, we investigated whether EGFR is required for *C*. *albicans* to induce the sustained activation of EphA2. We found that when EGFR was either knocked down with siRNA or inhibited with the specific EGFR kinase inhibitor, gefitinib [16], *C*. *albicans-*induced phosphorylation of EphA2 was transient, occurring within 30 min of infection, but declining to basal levels by 90 min (Figs 1A and S1A–S1C). These results suggest that in response to *C*. *albicans* infection, EphA2 phosphorylation is maintained by the activation of EGFR.

Cross talk between EphA2 and EGFR may reflect a physical interaction between these two receptors. To investigate this possibility, we used a proximity ligation assay (PLA), which forms a fluorescent spot when two proteins are located within 40 nm of each other [17]. In uninfected epithelial cells, the consistent PLA signal indicated that EphA2 associates with EGFR under basal conditions (Fig 1B, left panel). In epithelial cells infected with *C*. *albicans*, there was a strong PLA signal around some of the organisms, indicating an interaction between the two receptors. (Fig 1B, right panel). This aggregation appeared to be due to the movement of existing EphA2-EGFR complexes because the PLA signal was unchanged by the presence of *C*. *albicans*. As a control, we used the PLA to assess the association of EphA2 with HER2, which also interacts with *C*. *albicans* [5]. We found that there was low-level association between these two receptors that was unchanged by the presence of *C*. *albicans* (S2A Fig). The physical association between EphA2 and EGFR was verified by immunoprecipitation experiments, which showed that EphA2 and EGFR were co-immunoprecipitated from lysates of both uninfected and *C*. *albicans* infected epithelial cells (S2B Fig). Collectively, these results indicate that EphA2 and EGFR likely form part of a complex that enables each receptor to influence the activity of the other.

Because EphA2 and EGFR appeared to be mutually dependent, we investigated whether these two receptors influence the localization of each other on the epithelial cell surface. Knockdown of EphA2 by siRNA reduced the levels of both total and cell surface expressed EphA2, but had no effect on the levels of total and cell surface expressed EGFR (Figs 1C and S3). By contrast, knockdown of EGFR not only reduced the levels of total and cell surface expressed EGFR, but also decreased the amount of surface expressed EphA2, while leaving the total EphA2 levels unchanged (Figs 1C and S3). These results indicate that although the surface expression of EGFR is independent of EphA2, maximal expression of EphA2 on the epithelial cell surface is dependent on EGFR.

### Als3-mediated endocytosis enhances the targeting of candidalysin

**Fig 2:**
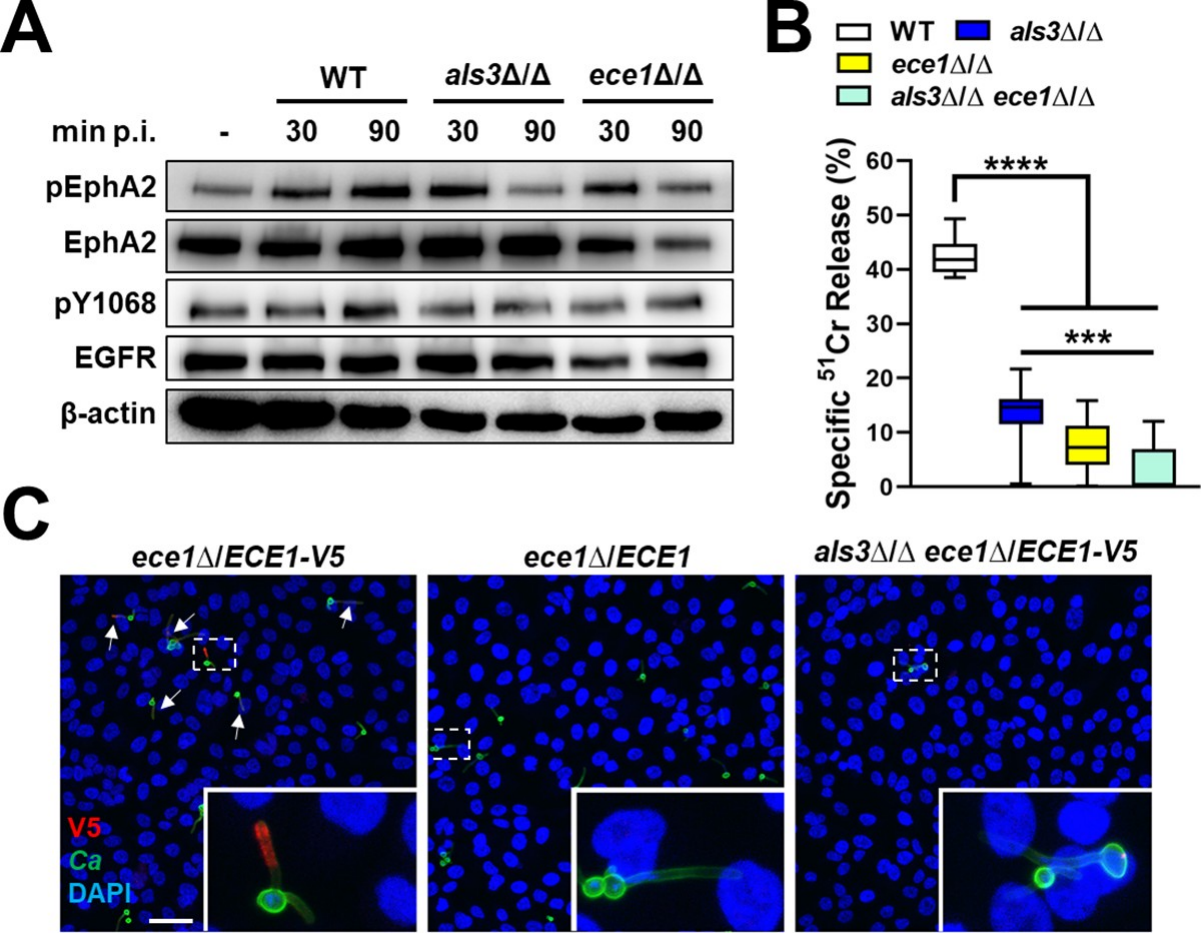
Als3 potentiates the targeting of candidalysin. (A) Immunoblot analysis showing the effects of the indicated *C*. *albicans* strains on the phosphorylation of EphA2 and EGFR after 30 and 90 min post-infection (p. i.). Results are representative a 3 independent experiments. Densitometric quantification of all 3 immunoblots such as the one in (A) is shown in S4 Fig. (B) Extent of oral epithelial cell damage caused by the indicated strains after 8 h of infection. Box whisker plots show median, interquartile range, and range of 3 independent experiments, each performed in triplicate. Statistical significance was determined by the Kruskal-Wallis test corrected for multiple comparisons. ***, *p* < 0.001; ****, *p* < 0.0001. Confocal microscopic images of oral epithelial cells after 90-min infection with candidalysin-V5 expressing strains of *C*. *albicans*. Arrows indicate endocytosed *C*. *albicans* hyphae with detectable candidalysin-V5. Boxes indicate the areas magnified in the insets. Scale bar: 40 μm.

Both the Als3 invasin and the candidalysin toxin are required for *C*. *albicans* to damage oral epithelial cells and activate EGFR [3,7,14,18]. To determine whether these virulence factors are required to sustain EphA2 activation, we constructed *als3*Δ/Δ and *ece1*Δ/Δ deletion mutants that were otherwise isogenic. While the wild-type strain stimulated phosphorylation of EGFR and induced prolonged phosphorylation of EphA2, both mutants failed to induce phosphorylation of EGFR and induced only transient phosphorylation of EphA2 (Figs 2A and S4). These results support the model that EGRF activation is required to sustain activation of EphA2 during *C*. *albicans* infection.

To investigate the relationship between Als3 and candidalysin in the capacity of *C*. *albicans* to damage and stimulate epithelial cells, we constructed an *als3*Δ/Δ *ece1*Δ/Δ double mutant. While this mutant had the same epithelial cell adherence and invasion defect as the *als3*Δ/Δ single mutant (S5 Fig), it had a greater epithelial cell damage defect, causing significantly less damage than the *als3*Δ/Δ single mutant (Fig 2B). One potential explanation for these results is that Als3-mediated epithelial cell invasion is required for maximal epithelial cell damage induced by candidalysin.

To test this hypothesis, we constructed an allele of *ECE1* that specified candidalysin with a C-terminal V5 epitope tag (S6 Fig). We then integrated a single copy of this allele into its native locus in the *ece1*Δ/Δ single mutant and the *als3*Δ/Δ *ece1*Δ/Δ double mutant. As a control, we integrated a single copy of an untagged *ECE1* allele into these strains at the same locus. We verified that the V5-tagged candidalysin (candidalysin-V5) was secreted by probing dot blots of concentrated culture supernatants with an anti-V5 antibody (S7A Fig). We also confirmed that the candidalysin-V5 was functional by demonstrating that complementation of the *ece1*Δ/Δ mutant with either a single copy of wild-type *ECE1* or *ECE1-V5* restored damage to approximately 50% of wild-type levels (S7B Fig).

To determine the localization of candidalysin during epithelial cell infection, we used confocal microscopy. We observed that there was intense staining of candidalysin-V5 around regions of *C*. *albicans* hyphae that were endocytosed by the epithelial cells (Fig 2C). By contrast, there was only weak staining around regions of the hyphae that remained outside of the epithelial cells. When the epithelial cells were infected with the *als3*Δ/Δ *ece1*Δ/E*CE1-V5* strain, very few hyphae were endocytosed and there was only weak staining of candidalysin-V5 (Fig 2C). There was no staining of candidalysin in cells infected with strains of *C*. *albicans* that did not contain the candidalysin-V5 construct (S7C Fig). These data indicate that Als3 promotes the targeting of candidalysin to host cells by inducing the formation of an endocytic vacuole in which candidalysin accumulates.

### *C*. *albicans* cells deficient in Als3 and candidalysin induce different patterns of EGFR activation, but similar inflammatory responses

**Fig 3:**
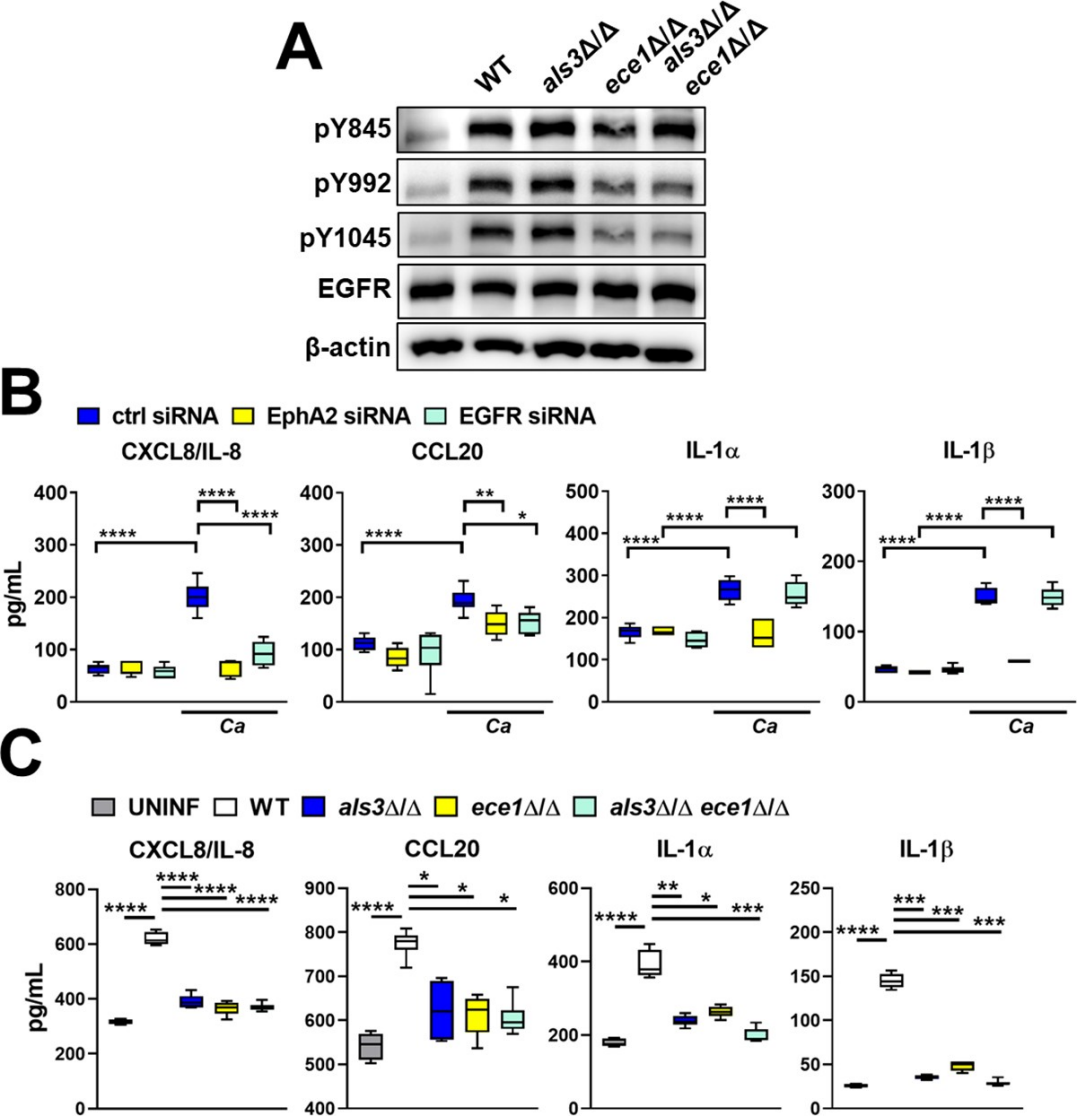
Effects of Als3 and Ece1 on EGFR signaling and induction of a pro-inflammatory response *in vitro*. (A) Immunoblot showing the phosphorylation of EGFR on tyrosines 845, 992, and 1045 in response to 90-min infection of oral epithelial cell with the indicated strains of *C*. *albicans*. Results are representative 3 independent experiments. Densitometric quantification of all 3 immunoblots such as the one in (A) is shown in S8A Fig. (B) Effects of siRNA knockdown of EGFR and EphA2 on the production of the indicated cytokines and chemokines in response to 8 h of infection with *C*. *albicans* (Ca). (C) Effects of 8 h infection of oral epithelial cells with the indicated strains of *C*. *albicans* on secretion of the indicated cytokines and chemokines. Data were analyzed using the Kruskal-Wallis test corrected for multiple comparisons. *, *p* < 0.05; **, *p* < 0.01; ***, *p* < 0.001; ****p, < 0.0001.

*C*. *albicans* infection induces the autophosphorylation of EGFR on multiple tyrosine residues in its intracytoplasmic tail [19]. As shown in Fig 2A, both Als3 and candidalysin are required for *C*. *albicans* to stimulate EGFR phosphorylation on Y1068. To gain a more comprehensive view of the patterns of EGFR phosphorylation induced by Als3 and candidalysin, we analyzed the phosphorylation of Y845, Y992, and Y1045 in epithelial cells that were infected for 90 min with the *als3*Δ/Δ, *ece1*Δ/Δ, and *als3*Δ/Δ *ece1*Δ/Δ mutants. Consistent with previous results [10,19], the wild-type strain induced EGFR phosphorylation at all three sites (Figs 3A and S8A). Although the *als3*Δ/Δ mutant failed to induce the phosphorylation of Y1068 (Fig 2A), it stimulated phosphorylation of the other tyrosine residues similarly to the wild-type strain (Figs 3A and S8A). Both the *ece1*Δ/Δ single mutant and the *als3*Δ/Δ *ece1*Δ/Δ double mutant induced significantly less phosphorylation of Y992 and Y1045 but induced wild-type levels of phosphorylation of Y845 (Figs 3A and S8A). Of note, the *als3*Δ/Δ+*ALS3* and *ece1*Δ/Δ+*ECE1* complemented strains induced wild-type levels of phosphorylation to Y1068 (S8B Fig). Collectively, these results indicate that Als3 is required to induce a much narrower pattern of EGFR activation than candidalysin.

Next, we compared the roles of EphA2 and EGFR in stimulating an epithelial cell proinflammatory response to wild-type *C*. *albicans*. We found that siRNA knockdown of EphA2 reduced the secretion of CXCL8/IL-8, CCL20, IL-1α, and IL-1β (Fig 3B). Although knockdown of EGFR inhibited the secretion of CXCL8/IL-8, CCL20, it had no effect on the release of IL-1α, and IL-1β (Fig 3B). Inhibition of EGFR phosphorylation with gefitinib had a similar effect (S9A Fig). These results suggest that activation of both EphA2 and EGFR is required for *C*. *albicans* to induce epithelial cells to secrete CXCL8/IL-8 and CCL20. They also indicate that EphA2, but not EGFR is required for *C*. *albicans* to stimulate the production of IL-1α and IL-1β.

Using mutant strains of *C*. *albicans*, we investigated the roles of Als3 and Ece1 in stimulating oral epithelial cells *in vitro*. Infection with the *als3*Δ/Δ and *ece1*Δ/Δ single mutants induced significantly less secretion of CXCL8/IL-8, CCL20, IL-1α, and IL-1β than the wild-type parent strain (Fig 3C). Secretion of these inflammatory mediators was restored to wild-type levels when the epithelial cells were infected with the *als3*Δ/Δ+*ALS3* and *ece1*Δ/Δ+*ECE1* complemented strains (S9B Fig). Infection with the *als3*Δ/Δ *ece1*Δ/Δ double mutant also failed to increase the production of inflammatory mediators, and the epithelial cell stimulation defect of this mutant was no greater than that of either the *als3*Δ/Δ or *ece1*Δ/Δ single mutants (Fig 3C). Collectively, these results are consistent with our model that Als3-mediated epithelial cell invasion enhances the targeting of candidalysin to the host cell, leading to activation of EphA2 and EGFR and stimulating secretion of CXCL8/IL-8 and CCL20. These results also suggest that this functional interaction between Als3 and candidalysin induces the production of IL-1α and IL-1β via a mechanism that is independent of EGFR.

### *C*. *albicans* hyphae stabilize activated EphA2 and EGFR in epithelial cells

**Fig 4:**
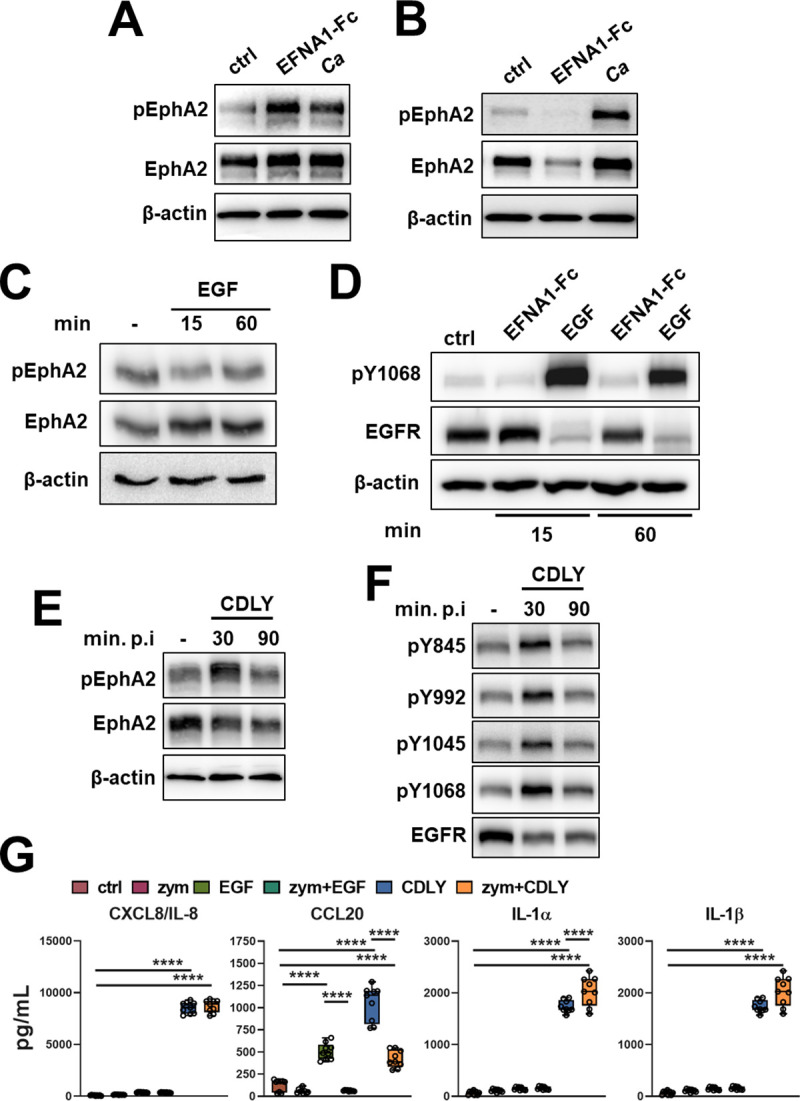
Soluble EphA2 and EGFR ligands induce a different epithelial cell response than *C*. *albicans*. (A and B) Immunoblots showing effects of 1 μg/ml ephrin A1-Fc (EFNA1-Fc) or yeast-phase *C*. *albicans* SC5314 (Ca) on the phosphorylation of ephrin type-A receptor 2 (EphA2) in OKF6/TERT-2 oral epithelial cells after stimulation for 15 min (A) and 60 min (B). (C and D) Effects of EFNA1-Fc and 100 ng/ml epidermal growth factor (EGF) on the phosphorylation and total cell levels of EphA2 (C) and EGFR (D). (E and F) Effects of 70 μM candidalysin (CDLY) on the phosphorylation and total cell levels of EphA2 (E) and EGFR (F) Results in A-F are representative of 3 independent experiments. Densitometric quantification of all 3 immunoblots is shown in S10A–S10F Fig. (G) Effects of 8-h incubation with EFNA1-Fc, EGF, zymosan (zym), and candidalysin on the epithelial cell production of the indicated cytokines and chemokines.

The native ligands of EphA2 and EGFR are ephrin A1 (EFNA1) and epidermal growth factor (EGF), respectively. These native ligands not only active their receptors, but also cause them to be internalized and traffic to the late endosome where they are rapidly degraded [20,21]. Because soluble and cell-associated ligands have different effects on receptor activation [22], we compared the epithelial cell response to prolonged exposure to EFNA1 and EGF versus *C*. *albicans*. We confirmed that exposure to EFNA1 and EGF stimulated the phosphorylation of their respective receptors and then induced receptor degradation by 60 min (Figs 4A–4D and S10A–S10D). By contrast, infection with *C*. *albicans* induced sustained receptor phosphorylation while maintaining stable levels of these receptors (Figs 2A, 4A and 4B and S4B), suggesting that the organism prevents receptor degradation.

Next, we analyzed the response of oral epithelial cells to recombinant candidalysin. We found that unlike viable *C*. *albicans*, candidalysin stimulated the phosphorylation of EphA2 at 30 min, but not at 90 min (Figs 4E and S10E). Also, exposure to candidalysin for 90 min caused a significant reduction in total EphA2 levels. Similarly, candidalysin induced phosphorylation of EGFR on tyrosines Y845, Y992, Y1045, and Y1068 at 30 min, but only induced phosphorylation of Y1068 at 90 min (Figs 4F and S10E). It reduced total EGFR levels after both 30 and 90 min. Thus, although candidalysin activates both EphA2 and EGFR, it induces a response that is similar to other soluble agonists and different from that induced by viable *C*. *albicans*.

To determine whether stimulating EphA2 and EGFR with soluble and/or particulate agonists altered the epithelial cell pro-inflammatory response, we stimulated oral epithelial cells with specific EphA2 and EGFR agonists, either alone or in combination, and then measured the release of CXCL8/IL-8, CCL20, IL-1α and IL-1β. Consistent with previous reports [10,15], incubating the cells with the particulate EphA2 agonist, zymosan did not significantly induce the production of any of these pro-inflammatory mediators (Fig 4G). Incubating the cells with the EGFR agonist, epidermal growth factor (EGF) increased the secretion of CCL20, but not the other cytokines. Candidalysin by itself strongly stimulated the production of all 4 pro-inflammatory mediators. Unexpectedly, when the cells were incubated with both zymosan and EGF, the secretion of CCL20 was reduced to basal levels. When the cells were incubated with zymosan and candidalysin, CCL20 production was inhibited and IL-1α production was increased relative to cells incubated with candidalysin alone. These data suggest that activation of EphA2 and EGFR with soluble and particulate agonists induces a different pro-inflammatory response in epithelial cells than is induced by intact, viable *C*. *albicans*.

### The response of OKF6/TERT-2 epithelial cells to *C*. *albicans* differs significantly from TR146 epithelial cells

**Fig 5:**
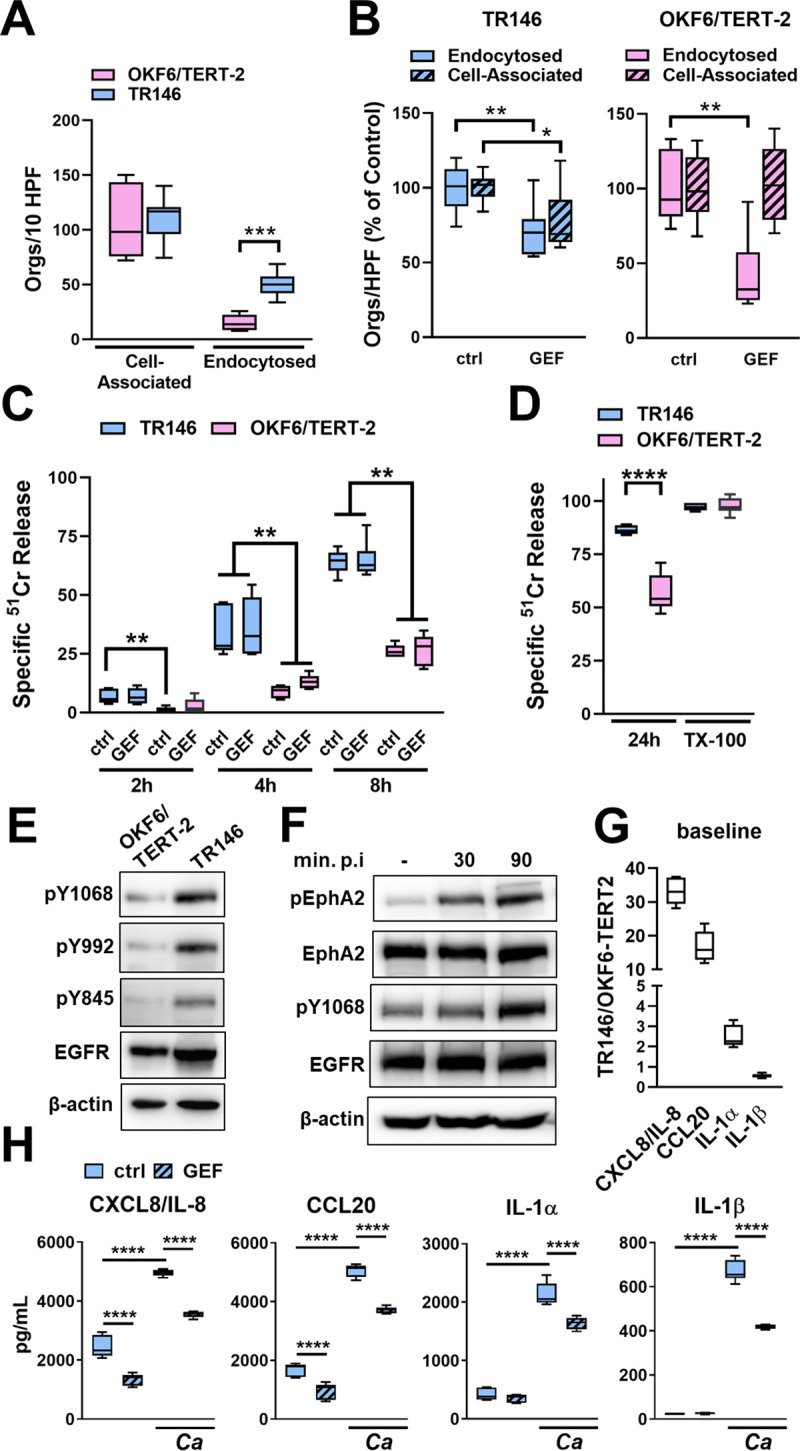
Comparison of the response of two oral epithelial cell lines to *C*. *albicans* infection and EGFR inhibition. (A) Adherence (cell-association) and endocytosis of wild-type *C*. *albicans* strain SC5314 by OKF6/TERT-2 and TR146 oral epithelial cell lines. (Orgs/10 HPF, organisms per 10 high-power fields) (B) Comparison of the effects of gefitinib (GEF) versus diluent (ctrl) on *C*. *albicans* adherence to and endocytosis by OKF6-TERT-2 and TR146 cells. (C) Time course of *C*. *albicans*-induced damage to OKF6-TERT-2 and TR146 cells. (D) Extent of damage to OKF6/TERT-2 and TR146 cells caused by *C*. *albicans* and Triton X-100 (TX-100) after 24 h incubation. (E) Comparison of the extent of phosphorylation on the indicated tyrosine residues of EGFR in unstimulated OKF6/TERT-2 and TR146 cells. (F) Phosphorylation of EphA2 and EGFR in TR146 cells after 30 and 60 min post-infection (p.i.) with *C*. *albicans*. Results in (E and F) are representative immunoblots from 3 independent experiments. Densitometric quantification of all 3 immunoblots is shown in S11 Fig. (G) Comparison of the basal release of the indicated inflammatory mediators by uninfected OKF6-TERT-2 and TR146 cells. Results are the ratio of TR146 cells to OKF6/TERT2 cells. (H) Effects of gefitinib on *C*. *albicans*-induced production of the indicated pro-inflammatory mediators by TR146 cells. Graphs show the results of 3 experiments, each performed in triplicate (A-D) or duplicate (G and H). The data in (A, B, and D) were analyzed with the Mann-Whitney test, and the data in (C, G, and H) were analyzed by the Kruskal-Wallis test corrected for multiple comparisons. *, *p* < 0.05; **, *p* < 0.01; ***, *p* < 0.001; ****p, < 0.0001.

The above experiments were conducted with OKF6/TERT-2 oral epithelial cells, which were generated by transfecting normal human oral keratinocytes with a constitutively active telomerase [23]. Using the TR146 human buccal squamous cell carcinoma cell line, Ho et al. also found that *C*. *albicans-*induced activation of EGFR is required for the fungus to stimulate oral epithelial cells to secrete multiple cytokines [14]. One difference between their results and those presented here is that EGFR was required for induction of IL-1α and IL-1β in TR146 cells but not OKF6/TERT-2 cells. To investigate this difference, we compared the interactions of *C*. *albicans* with both cell lines. We found that while the adherence of *C*. *albicans* to both types of epithelial cells was similar, TR146 cells endocytosed over 3-fold more *C*. *albicans* cells than did OKF6/TERT-2 cells (Fig 5A). Also, inhibition of EGFR with gefitinib modestly reduced the adherence of *C*. *albicans* to TR146 cells, but not OKF6/TERT-2 cells and decreased *C*. *albicans* endocytosis by TR146 cells by 30% and endocytosis by OKF6/TERT-2 cells by 64% (Fig 5B). Thus, in TR146 cells relative to OKF6/TERT-2 cells, EGFR is more important for adherence but less important for inducing the endocytosis of *C*. *albicans*.

In a time-course study, *C*. *albicans* caused significantly more damage to TR146 cells than to OKF6/TERT-2 cells at all time points (Fig 5C). In fact, after 24 h of infection, *C*. *albicans-*induced damage of TR146 cells was near-maximal, whereas the extent of damage to the OKF6/TERT-2 cells was significantly less (Fig 5D). Gefitinib had no effect on the extent of *C*. *albicans*-induced damage to either cell line (Fig 5C). Collectively, these data indicate that TR146 cells are more susceptible to *C*. *albicans*-induced damage than OKF6/TERT-2 cells.

**Fig 6:**
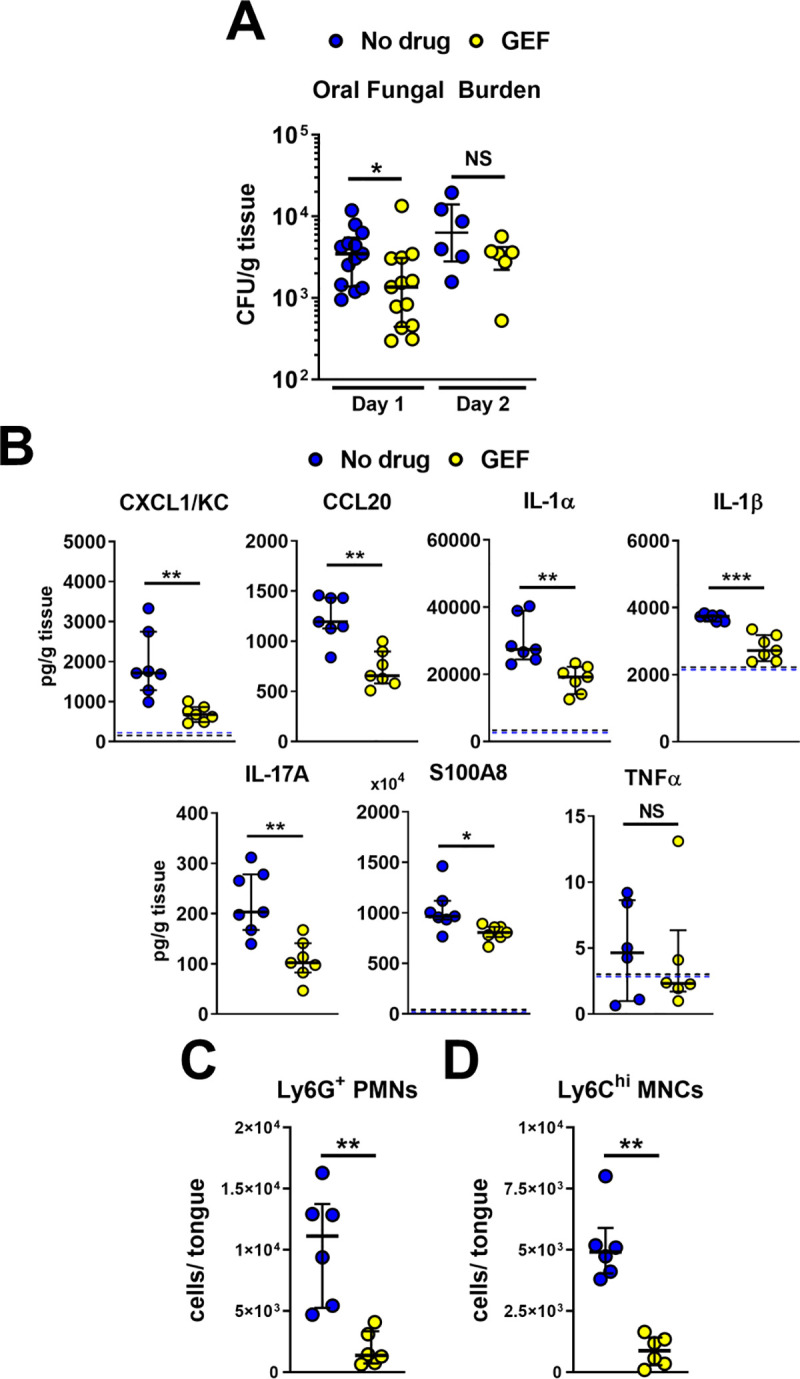
Pharmacological inhibition of EGFR decreases early fungal burden and reduces the inflammatory response. (A) Oral fungal burden of control (no drug) and gefitinib (GEF) treated mice after 1 d post-infection with wild-type *C*. *albicans* strain SC5314. Results are the median ± interquartile range of at total of 14 mice per group from two independent experiments. The y-axis is set at the limit of detection. (B) Levels of the indicated inflammatory mediators in the oral tissues of the mice. Results are from a total of 7 mice per group from two independent experiments. Dashed line indicates the median level of inflammatory mediators in uninfected mice if above 0. (C and D) Levels of neutrophils (C) and inflammatory monocytes (D) in the tongues of control and gefitinib-treated mice after 1 d of infection. Results are from a total of 6 mice per group from two independent experiments. Statistical significance in (A, C and D) was determined using the Mann-Whitney test and in (B) by the Kruskal-Wallis test corrected for multiple comparisons. NS, not significant; *, *p* < 0.05; **, *p* < 0.01.

The extent of EGFR phosphorylation and cytokine production by TR146 cells and OKF6/TERT-2 cells also differed. Uninfected TR146 cells had significantly higher levels of total and phosphorylated EGFR than OKF6/TERT-2 cells (Figs 5E and S11A). When TR146 cells were infected with *C*. *albicans*, there was increased phosphorylation of EphA2 and EGFR, similarly to what was observed with OKF6/TERT-2 cells (Figs 5F and S11B). Uninfected TR146 cells also secreted over 30-fold more CXCL8/IL-8, 15-fold more CCL20, and 2-fold more IL-1α than OKF6/TERT-2 cells (Fig 5G). The levels of these cytokines and IL-1β significantly increased when the TR146 cells were infected with *C*. *albicans* (Fig 5H). While gefitinib inhibited the production of CXCL8/IL-8 and CCL20 by *C*. *albicans* infected TR146 cells, it also decreased the production of IL-1α and IL-1β (Fig 6H). Thus, EGFR governs *C*. *albicans*-induced production of chemokines in both cell lines, but it regulates production of IL-1α and IL-1β only in TR146 cells.

### Inhibition of EGFR decreases EphA2 phosphorylation, oral fungal burden, and the host inflammatory response during OPC

To assess the role of EGFR in mediating the host inflammatory response to *C*. *albicans* during OPC, we treated immunocompetent mice with gefitinib and then orally inoculated them with *C*. *albicans*. Immunocompetent mice were used to avoid potentially confounding effects of immunosuppression on the host inflammatory response. When immunocompetent mice are orally inoculated with *C*. *albicans* strain SC5314, infection persists for 2 days, after which the organism is cleared by the host inflammatory response [13,24].

Using flow cytometry and phosphospecific antibodies, we assessed the effects of gefitinib on EGFR and EphA2 phosphorylation in the oral epithelium. As predicted by the *in vitro* data, treatment with gefitinib reduced the phosphorylation of both EGFR and EphA2 in CD45^-^ EpCam^+^ cells after 1 d of infection (S12A–S12D Fig). At this time point, the gefitinib-treated mice had a 3-fold reduction in oral fungal burden compared to untreated mice (Fig 6A), probably due to inhibition of epithelial cell invasion by the drug.

**Fig 7:**
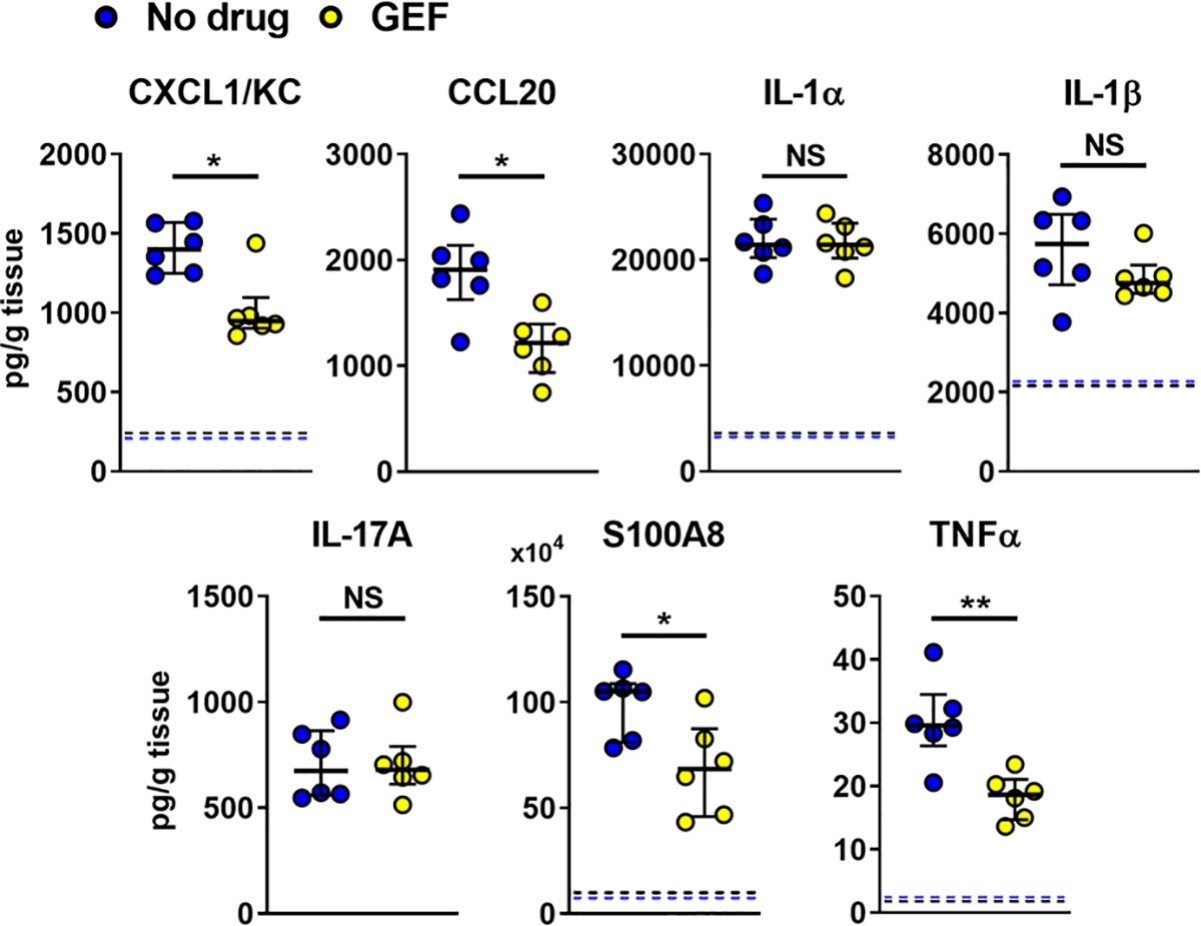
Pharmacological inhibition of EGFR decreases reduces a subset of the inflammatory response at day 2 post-infection. (A) Levels of the indicated proinflammatory mediators in the oral tissues of control (no drug) and gefitinib (GEF) treated mice after 2 d post-infection with wild-type *C*. *albicans*. Results are the median ± interquartile range of a total of 6 mice per group from a single experiment. Dashed line indicates the median level of inflammatory mediators in uninfected mice if above 0. Statistical significance was determined using the Mann-Whitney test. NS, not significant; *, *p* < 0.05; **, *p* < 0.01.

To determine the effects of gefitinib on the inflammatory response, we measured whole tongue levels of CXCL1/KC, CCL20, IL-1α and IL-1β. We also measured the levels of IL-17A, S100A8, and TNFα o obtain a more comprehensive view of the effects of gefitinib. After 1 day of infection, the oral tissues of the gefitinib-treated mice contained significantly less CXCL1/KC, CCL20, IL-1α, IL-1β, IL-17A, and the host defense peptide S100A8 than control mice (Fig 6B). There were also significantly fewer neutrophils and inflammatory monocytes in the oral tissues of the gefitinib-treated mice (Figs 6C and 6D and S12E). After 2 days of infection, the oral fungal burden of the gefitinib-treated mice was similar to the control mice (Fig 6A). At this time point, treatment with gefitinib reduced the levels of CXCL1/KC, CCL20, S100A8 and TNFα, but not IL-1α, IL-1β, and IL-17A (Fig 7). These results indicate that blocking EGFR reduces a subset of the early inflammatory response to acute OPC in immunocompetent mice.

Because gefitinib impaired the host inflammatory response to *C*. *albicans*, we investigated its effects on the candidacidal activity of neutrophils and macrophages. We found that gefitinib did not decrease the capacity of human neutrophils, bone marrow (BM) neutrophils isolated from gefitinib-treated mice, or murine BM derived macrophages to kill *C*. *albicans in vitro* (S13 Fig). Taken together, these results indicate that while EGFR signaling is required for epithelial cells to mount a pro-inflammatory response to *C*. *albicans*, it is dispensable for governing phagocyte killing of the organism.

### *C*. *albicans* adhesins/invasins and candidalysin are required for maximal virulence during OPC

**Fig 8:**
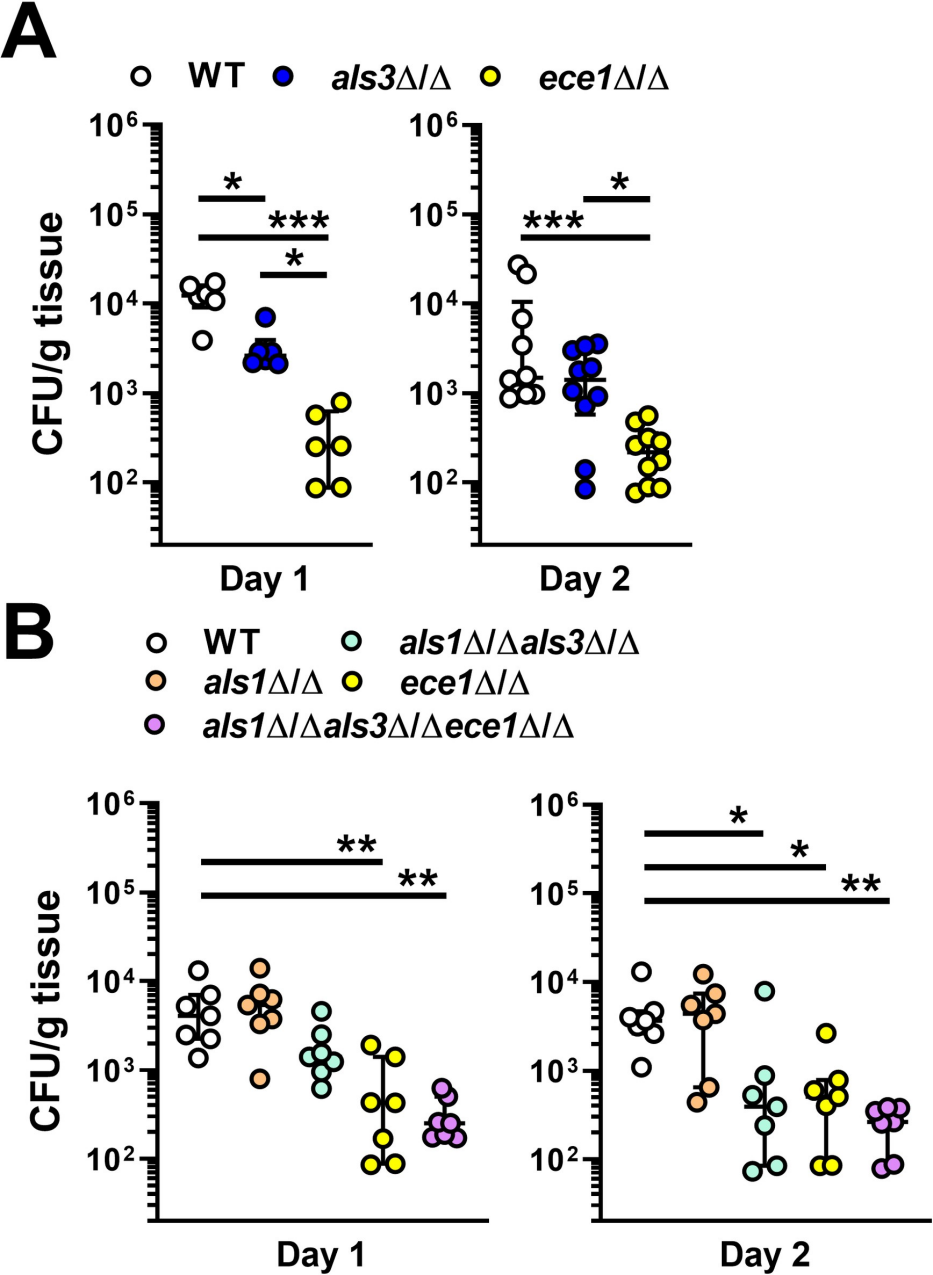
Roles of Als1, Als3, candidalysin in *C*. *albicans* virulence during OPC. (A and B) Oral fungal burden of Balb/c mice 1 and 2 d after inoculation with the indicated strains of *C*. *albicans*. The WT strain was SC5314. Results are the median ± interquartile range of 6–8 mice per strain from single experiments, except for d 2 in (A), which are from a total of 10 mice from 2 independent experiments. The data were analyzed by the Kruskal-Wallis test corrected for multiple comparisons. *, *p* < 0.05; **, *p* < 0.01; ***, *p* < 0.001.

To investigate the roles Als3 and candidalysin in stimulating the host response during oropharyngeal candidiasis, we orally infected immunocompetent mice with *C*. *albicans als3*Δ/Δ and *ece1*Δ/Δ mutants. After 1 day of infection, mice inoculated with the *als3*Δ/Δ mutant had reduced oral fungal burden relative to those infected with the wild-type strain, and mice inoculated with the *ece1*Δ/Δ mutant had an even greater decrease in fungal burden (Fig 8A). After 2 days of infection, the fungal burden of mice infected with the *als3*Δ/Δ mutant was similar to that of mice infected with the wild-type strain, whereas the fungal burden of mice infected with the *ece1*Δ/Δ mutant remained significantly lower (Fig 8A). We also determined that C57BL/6 mice infected with the *ece1*Δ/Δ mutant constructed in either strain SC5314 or BWP17 had reduced oral fungal burden (S14 Fig). Thus, in the acute oropharyngeal candidiasis model, Als3 is necessary for maximal infection at day 1, whereas candidalysin is required for maximal infection at both days 1 and 2.

Although the *als3*Δ/Δ mutant is highly defective in invading oral epithelial cells *in vitro*, the oral fungal burden data suggest that this mutant has only a modest invasion defect *in vivo*. To further reduce the capacity of *C*. *albicans* to invade the oral epithelium *in vivo*, we constructed an *als1*Δ/Δ *als3*Δ/Δ double mutant. Our rationale was that *C*. *albicans* must first adhere to host cells in order to invade them. *C*. *albicans* Als1 is structurally similar to Als3 and can function as both an adhesin and invasin [3,25]. Therefore, the presence of Als1 in the *als3*Δ/Δ mutant could enable the organism to adhere to and invade the oral epithelium in the absence of Als3. To test this hypothesis, we constructed *als1*Δ/Δ, *als1*Δ/Δ *als3*Δ/Δ, and *als1*Δ/Δ *als3*Δ/Δ *ece1*Δ/Δ mutants. *In vitro*, the *als1*Δ/Δ *als3*Δ/Δ mutant had significantly reduced adherence to OKF6/TERT-2 cells compared to the *als3*Δ/Δ mutant, indicating that Als1 and Als3 make independent contributions to *C*. *albicans* adherence (S15A Fig). Because the *als3*Δ/Δ single mutant was endocytosed so poorly by oral epithelial cells and caused so little host cell damage, deletion of *ALS1* did not reduce these interactions further (S15B and S15C Fig)

After 1 day of oral infection, mice infected with the *als1*Δ/Δ *als3*Δ/Δ mutant had a slight reduction in oral fungal burden relative to mice infected with the wild-type strain, but this difference was not statistically significant (Fig 8B). At this time point, the fungal burden of mice infected with *ece1*Δ/Δ single mutant and the *als1*Δ/Δ *als3*Δ/Δ *ece1*Δ/Δ triple mutant was significantly reduced. After 2 days of infection, the fungal burden of mice infected with the *als1*Δ/Δ *als3*Δ/Δ double mutant was decreased to the same extent as mice infected with either the *ece1*Δ/Δ single mutant or the *als1*Δ/Δ *als3*Δ/Δ *ece1*Δ/Δ triple mutant (Fig 8B). These data suggest that adherence and invasion mediated by Als1 and Als3 combined with host cell damage caused by candidalysin play key roles in inducing acute OPC.

**Fig 9:**
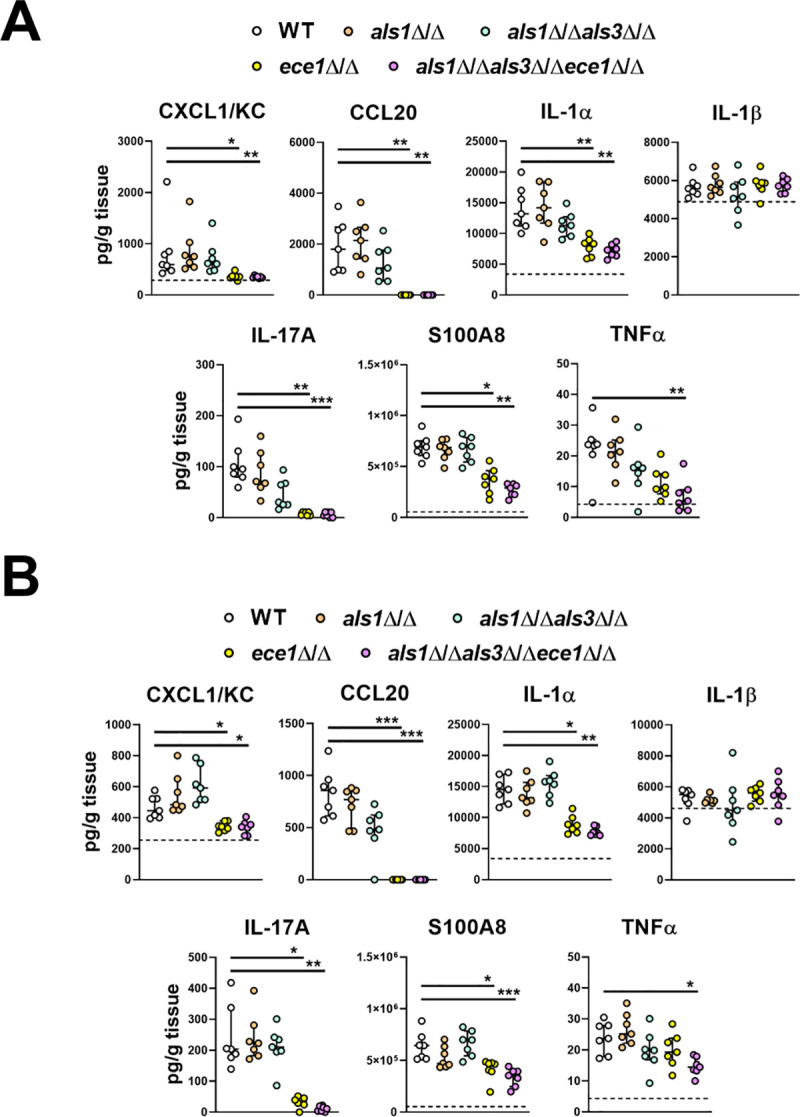
Induction of the oral inflammatory response by *C*. *albicans* invasins and candidalysin. Levels of the indicated inflammatory mediators in the tongues of mice orally inoculated with the indicated strains of *C*. *albicans* after 1 d (A) and 2 d (B) of infection. Results are the median ± interquartile range of a total of 7 mice in each group from a single experiment. Median levels of inflammatory mediators in uninfected mice are indicated with dashed line if above 0. The data were analyzed by the Kruskal-Wallis test corrected for multiple comparisons. *, *p* < 0.05; **, *p* < 0.01; ***, *p* < 0.001.

To determine the contributions of Als1, Als3 and candidalysin to the induction and maintenance of the oral innate immune response, we determined the levels of inflammatory mediators in the infected tongues after 1 and 2 days of infection. Three different patterns of response were observed (Fig 9). The levels of IL-1β were similar in mice infected with all strains of *C*. *albicans*, indicating that the production of this cytokine is independent of Als1, Als3 and candidalysin. The levels of many inflammatory mediators, such as IL-1α, CXCL1/KC, CCL20, IL-17A, and S100A8 were decreased to the same extent in mice infected with either the *ece1*Δ/Δ mutant or the *als1*Δ/Δ *als3*Δ/Δ *ece1*Δ/Δ mutant, suggesting that the production of these mediators is dependent on candidalysin but independent of Als1 and Als3. The levels of TNFα were significantly reduced only in mice infected with the *als1*Δ/Δ *als3*Δ/Δ *ece1*Δ/Δ mutant, signifying that Als1, Als3, and candidalysin function cooperatively to induce the production of this cytokine. Thus, while the production of some inflammatory mediators during OPC is largely driven by candidalysin, Als1 and Als3 contribute to the production of TNFα.

## Discussion

**Fig 10:**
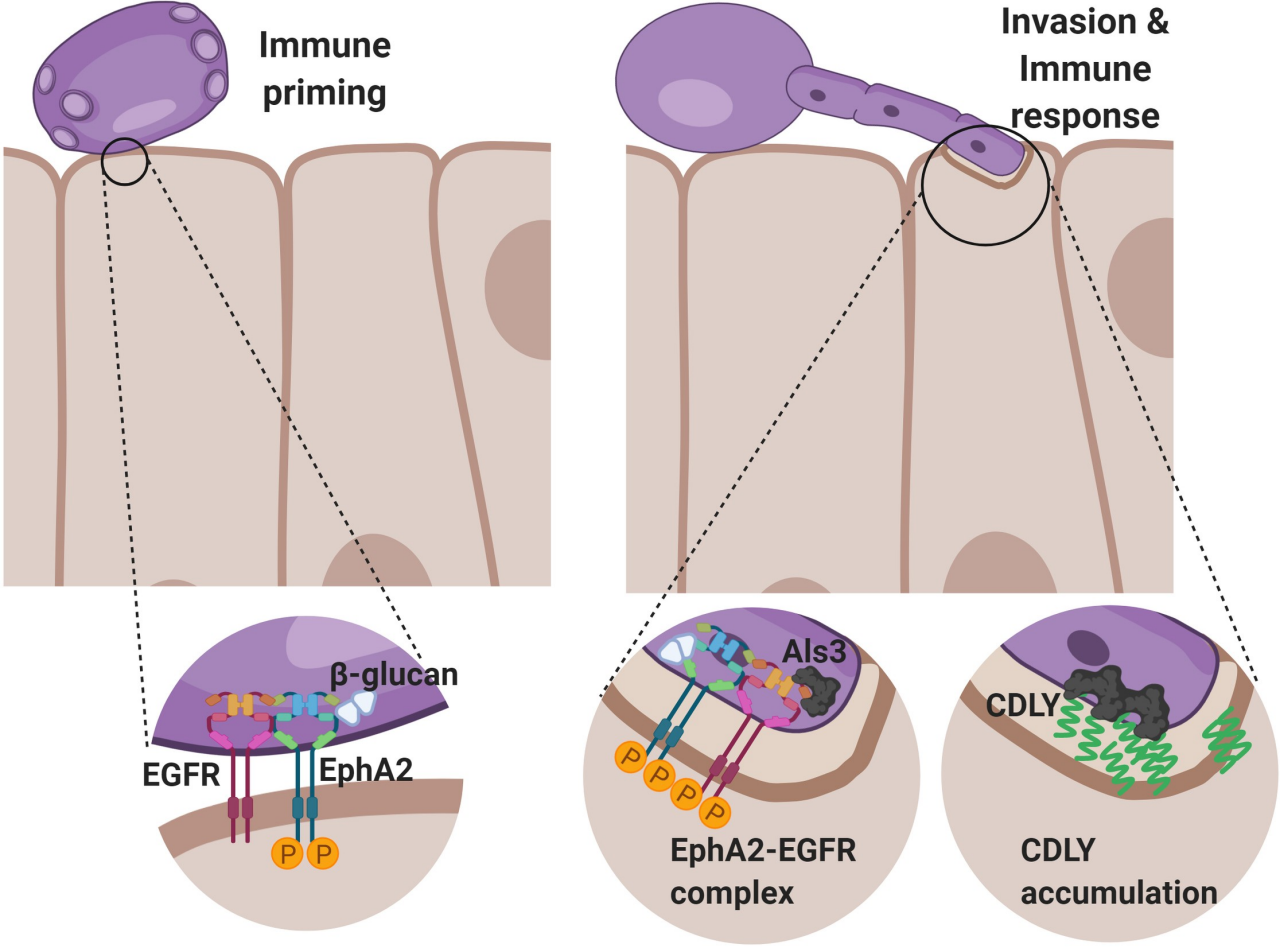
Model of the interactions among Als3, candidalysin, EphA2 and EGFR in *C*. *albicans* invasion of oral epithelial cells and induction of a pro-inflammatory response. (Left panel) Exposed β-glucan on the surface of a *C*. *albicans* cell interacts with and activates EphA2, priming the epithelial cell to respond to *C*. *albicans*. (Right panel) When the *C*. *albicans* cell germinates, it expresses Als3 and secretes candidalysin, which activate EGFR and prevent the EphA2-EGFR complex from being degraded. Als3 induces the epithelial cells to endocytose the hypha, leading to the formation of an endocytic vacuole in which the secreted candidalysin can accumulate. The higher concentration of candidalysin damages the epithelial cell and stimulates it to secrete pro-inflammatory cytokines and chemokines by both EGFR-dependent and EGFR-independent signaling pathways.

Oral epithelial cells play a central role in orchestrating the host defense against *C*. *albicans* during OPC [1,9,10,15,26]. Our current data show that two epithelial cell receptors, EphA2 and EGFR interact to sense the presence of *C*. *albicans* and initiate a pro-inflammatory response. Previously, we found that EphA2 activation is required for *C*. *albicans* to stimulate EGFR [10]. Here, we determined that activation of EGFR is in turn necessary for *C*. *albicans* to sustain activation of EphA2, both *in vitro* and *in vivo*. Also, the presence of EGFR is required for EphA2 to be expressed on the epithelial cell surface. The results of the PLA and immunoprecipitation experiments indicate that EphA2 and EGFR function as part of a complex, thus providing an explanation for how these two receptors function interdependently to mediate the epithelial cell response to *C*. *albicans* (Fig 10). Whether EphA2 and EGFR interact with each other directly or indirectly remains to be determined.

EphA2 and EGFR are known to interact in epithelial cell cancers, especially those that have become resistant to EGFR inhibitors, in which siRNA knockdown of EphA2 restores sensitivity to pharmacological EGFR inhibition [27,28]. Interestingly, treatment of malignant cell lines with soluble EFNA1 has the same effect as EphA2 siRNA, presumably because EFNA1 induces EphA2 endocytosis and subsequent degradation. We found that EGF activates EGFR and induces its degradation while recombinant candidalysin activates both EphA2 and EGFR and induces their degradation. By contrast, infection with viable *C*. *albicans* induces sustained activation of both receptors and prevents their degradation. The probable explanation for this result is that the interaction of *C*. *albicans* with the EphA2-EGFR complex anchors it to the cell membrane and prevents it from trafficking to the late endosome for degradation.

The current data indicate that Als3-mediated invasion enhances the targeting of candidalysin to host cells. We found that candidalysin accumulated around the internalize portion of *C*. *albicans* hyphae, indicating that Als3-mediated endocytosis enhances the targeting of candidalysin by creating a region of higher toxin concentration. This model is supported by multiple lines of data, including the findings that an *als3*Δ/Δ mutant has reduced capacity to damage oral epithelial cells and stimulate them to secret pro-inflammatory cytokines *in vitro*, that inhibiting epithelial cell endocytosis of *C*. *albicans* with cytochalasin D blocks fungal-induced damage [29], and that deletion of *ECE1* in the *als3*Δ/Δ mutant does not result in an additive reduction in epithelial cell damage or production of proinflammatory mediators. It has also been reported that the epithelial cell damage defect of an *als3*Δ/Δ mutant can be reversed if a very high inoculum is used [30]. Based on our results, the likely explanation for this finding is that at very high inocula, the concentration of candidalysin that is released is sufficient to cause epithelial cell damage even in the absence of an endocytic vacuole.

The phosphorylation of specific tyrosine residues on the intracytoplasmic domain of EGFR is necessary to induce the production of downstream pro-inflammatory mediators. A notable result was that although the *als3*Δ/Δ mutant induced a different pattern of tyrosine phosphorylation on EGFR relative to the *ece1*Δ/Δ mutant, both strains induced similar epithelial cell pro-inflammatory responses. When the epithelial cells were infected with the *als3*Δ/Δ mutant, there was reduced phosphorylation of Y1068 on EGFR, but the phosphorylation of Y845, Y992, and Y1045 was still induced. By contrast, the *ece1*Δ/Δ mutant induced significantly less phosphorylation of not only Y1068, but also of Y992 and Y1045. However, both the *als3*Δ/Δ and the *ece1*Δ/Δ mutant failed to induce the production of CXCL8/IL-8, CCL20, IL-1α, or IL-1β. Previously, we determined that treating oral epithelial cells with interferon-γ or an inhibitor of the aryl hydrocarbon receptor also blocked the *C*. *albicans-*induced phosphorylation of EGFR on Y1068 and reduced the endocytosis of the organism [19]. Taken together, these results suggest that phosphorylation of Y1068 on EGFR is likely the key event that triggers both the endocytosis of *C*. *albicans* and the production of a pro-inflammatory mediators by oral epithelial cells.

We found that *C*. *albicans* activation of EGFR resulted in the production of proinflammatory mediators by two different oral epithelial cell lines. Recently, it has been reported that candidalysin activates EGFR and stimulates the TR146 oral epithelial cell line to produce Il-1α, IL-1β, IL-6, G-CSF, and GM-CSF [14]. In the current work, knockdown of EGFR with siRNA or inhibition of EGFR with gefitinib significantly reduced *C*. *albicans*-induced production of CXCL8/IL-8 and CCL20 by both TR146 cells and OKF6-TERT2 cells. However, inhibition of EGFR only decreased production of IL-1α and IL-1β by TR146 cells, but not by OKF6-TERT2 cells. It was also determined that TR146 cells had higher basal levels of total and phosphorylated EGFR than OKF6-TERT2 cells, which may explain why cytokine production by TR146 cells was more sensitive to EGFR inhibition. Nevertheless, the results with both oral epithelial cells lines indicate that *C*. *albicans* activates EGFR, which induces the production of many proinflammatory mediators.

The central role of EGFR in mediating the host inflammatory response was demonstrated in the immunocompetent mouse model of OPC. Treatment of mice with gefitinib inhibited *C*. *albicans*-induced phosphorylation of both EphA2 and EGFR in the oral epithelial cells, and reduced the tissue levels of CXCL1/KC, CCL20, and S100A8 after both 1 and 2 days of infection. As a result, the accumulation of neutrophils and inflammatory monocytes in the oral tissues was dramatically decreased. In the gefitinib-treated mice, the levels of IL-1α, IL-1β, IL-17A, and TNFα were only reduced at a single time point, suggesting that the production of these cytokines may be governed independently of EGFR. Previously, we found that IL-1β levels were reduced in *EphA2*^-/-^ mice with OPC [10], and that activation of EphA2 with purified β-glucans is insufficient to stimulate the production of any cytokines. These two results suggest that EphA2 likely interacts with at least one additional host receptor, other than EGFR, to induce IL-1β production during OPC. Previously, we showed that *C*. *albicans* interacts with additional epithelial cell receptors, such as E-cadherin and the platelet-derived growth factor receptor B [31]. Whether these receptors interact with EphA2 remains to be determined.

Inhibition of EGFR has different effects in different models of mucosal infection. Recently, Ho et al. [14] reported that in the zebrafish swimbladder model of mucosal candidiasis, inhibition of EGFR with AG1478 impaired the accumulation of neutrophils and led to enhanced mortality, even though it had no effect on fungal burden. Our findings demonstrate that in immunocompetent mice, gefitinib has different effects; although it reduces cytokine production and accumulation of phagocytes, it also decreases oral fungal burden, lessening disease severity. As inhibition of EGFR reduces *C*. *albicans* endocytosis by oral epithelial cells *in vitro* [5], it is probable that the decrease in oral fungal burden by EGFR inhibition is due to impaired epithelial cell invasion *in vivo*. The capacity of EGFR inhibition to decrease oral fungal burden in immunocompetent mice [14] and in steroid-immunosuppressed mice [5] has been reported previously, but its effects on the host inflammatory response in mice was previously unknown.

When we tested the virulence of the *C*. *albicans* mutants with deletions in *ALS1*, *ALS3*, and/or *ECE1*, we found that some of the *in vitro* data were recapitulated in mice. After 2 days of infection, mice inoculated with the *ece1*Δ/Δ mutant, the *als1*Δ/Δ *als3*Δ/Δ mutant, and the *als1*Δ/Δ *als3*Δ/Δ *ece1*Δ/Δ mutant all had a similar reduction in oral fungal burden. This finding parallels the reduced epithelial cell damage induced by these strains *in vitro* and indicates that the combination of Als1 and Als3 function along with candidalysin to induce oral infection. It was notable that although the *als1*Δ/Δ *als3*Δ/Δ mutant had extremely impaired adherence to and invasion of oral epithelial cells *in vitro*, its virulence defect in mice was less severe. A likely explanation for this result is that other adhesins and invasins such as Hwp1 [32] and Ssa1 [4] may compensate for the absence of Als1 and Als3 *in vivo*. It is also possible that the *als1*Δ/Δ *als3*Δ/Δ mutant may invade oral epithelial cells *in vivo* by a receptor-independent mechanism, such as by active penetration [2].

The mouse data indicated that different subsets of the host inflammatory response are induced by different combinations of Als1, Als3, and candidalysin. Mice infected with the *ece1*Δ/Δ mutant had reduced levels of CXCL1/KC and CCL20 in their oral tissues, similar to mice that had been treated with gefitinib. Although mice infected with the *als1*Δ/Δ *als3*Δ/Δ *ece1*Δ/Δ mutant had a reduction in these chemokines comparable to mice infected with the *ece1*Δ/Δ mutant, animals infected with the *als1*Δ/Δ *als3*Δ/Δ mutant had wild-type levels. Collectively, these results suggest that candidalysin is necessary for *C*. *albicans* to active EGFR *in vivo*, which stimulates the production of CXCL1/KC and CCL20. These results also indicate that *in vivo*, Als1 and Als3 are dispensable for the induction of this aspect of the inflammatory response.

The data with the mutant strains of *C*. *albicans* showed that Als1, Als3, and candidalysin stimulate some proinflammatory responses independently of EGFR. While mice treated with gefitinib only had reduced levels of IL-1α and IL-17A at day 1 post infection, mice infected with either the *ece1*Δ/Δ mutant or the *als1*Δ/Δ *als3*Δ/Δ *ece1*Δ/Δ mutant had reduced levels of these cytokines at both days 1 and 2. Thus, at the later time point, candidalysin induces the production of IL-1α and IL-17A independently of EGFR. Also, while the levels of TNFα were not reduced in mice treated with gefitinib, they were consistently decreased only in mice that were infected with the *als1*Δ/Δ *als3*Δ/Δ *ece1*Δ/Δ mutant. These data suggest that the induction of TNFα production is independent of EGFR signaling, but dependent on the combined activity of Als1, Als3, and candidalysin.

The current results indicate that the capacity of candidalysin to induce IL-1β production is dependent on the anatomic site of infection. We found that mice infected with the *ece1*Δ/Δ mutant strains had wild-type levels of IL-1β in their oral tissues, indicating that production of this cytokine is independent of candidalysin. In the mouse model of disseminated candidiasis, candidalysin is dispensable for IL-1β production in the kidneys [33], but essential for IL-1β production in the brain [34]. Thus, *C*. *albicans* infection induces IL-1β production in the oral mucosa by a different mechanism than in the brain.

A paradoxical finding was that although mice infected with the *ece1*Δ/Δ mutant had a markedly reduced inflammatory response, they also had reduced oral fungal burden. This reduction in oral fungal burden was observed in two strains of mice infected with independent *ece1*Δ/Δ mutants and has also been reported in the immunosuppressed mouse model of OPC [7]. Because of the decreased inflammatory response induced by the *ece1*Δ/Δ mutant, this strain would be expected to proliferate unimpeded in the oral cavity. For example, when mice are infected intravenously with an *ece1*Δ/Δ mutant, there is significantly reduced production of proinflammatory cytokines and accumulation of neutrophils in the brain, and this dampened inflammatory response leads to increased brain fungal burden [34]. One potential explanation for the reduced oral fungal burden in mice infected with the *ece1*Δ/Δ mutant is that even in the absence of candidalysin, there is still some inflammatory response, possibly induced by IL-1β [35] and the residual phagocytes are able to clear the fungus. Alternatively, epithelial cell damage by candidalysin may release nutrients that are vital for fungal proliferation.

Overall, the data presented here indicate that EphA2, EGFR, Als1, Als3 and candidalysin cooperate in *C*. *albicans*-induced epithelial cell activation and damage during OPC (Fig 10). Although either Als3 or candidalysin is required for *C*. *albicans* to activate EGFR and stimulate epithelial cell secretion of proinflammatory mediators *in vitro*, induction of the oral inflammatory response *in vivo* relies predominantly upon candidalysin-induced activation of EGFR. It is also noteworthy that some inflammatory responses are induced independently of both candidalysin-and EGFR. It is possible that such responses are stimulated by other *C*. *albicans* invasins, including Ssa1 and/or additional epithelial cell receptors, such as E-cadherin, the aryl hydrocarbon receptor, and platelet-derived growth factor B [3,4,19,31]. Studies to delineate the roles of these additional fungal factors and epithelial cell receptors in stimulating the pro-inflammatory response to *C*. *albicans* are currently underway.

## Materials and methods

### Ethics statement

All animal work was approved by the Institutional Animal Care and Use Committee (IACUC) of the Lundquist Institute at Harbor-UCLA Medical Center. The collection of blood from human volunteers for neutrophil isolation was also approved by the Institutional Review Board of the Lundquist Institute at Harbor-UCLA Medical Center. Written informed consent was obtained from all subjects prior to phlebotomy.

### Fungal strains

The *C*. *albicans* strains used in this study are listed in S1 Table. For strain construction, a transient CRISPR-Cas9 system was employed [36]. Primers used for strain construction and confirmation are listed in S2 Table. To construct the initial *ece1*Δ/Δ and *als3*Δ/Δ mutants, MH216, a *his1*Δ/Δ NatR derivative of strain SC5314 was utilized [37]. To delete *ECE1*, MH216 was transformed with the Cas9 DNA cassette, ECE1-2 sgRNA DNA cassette, NAT1-5 sgRNA DNA cassette, and *ece1Δ*::*r1HIS1r1* repair template. *ALS3* was deleted in a similar manner, except that the ALS3-5P sgRNA DNA cassette and *als3Δ*::*r1HIS1r1* repair template were used. Transformants were selected on complete synthetic medium (CSM) plates without histidine, and screened for nourseothricin sensitivity on YPD + nourseothricin plates. His+ NatS transformants were checked for deletion of *ECE1* or *ALS3* by PCR genotyping using primers ECE1 check up/F and ECE1 check int/R, and primers ALS3 chk up/F and ALS3 chk int/R, respectively. The presence of the *r1HIS1r1* cassette was verified by PCR genotyping using primers ECE1 check up/F and CdHIS1 Check Int/R or primers ALS3 chk up/F T and CdHIS1 Check Int/R. To generate the *als3*Δ/Δ *ece1*Δ/Δ double mutant, the *als3*Δ/Δ mutant MH562 was independently transformed with the Cas9 DNA cassette, the ECE1-2 sgRNA DNA cassette, and the *ece1*Δ::*r3NAT1r3* repair template. Transformants were selected on YPD plates containing *400* μg/ml nourseothricin and NatR transformants were PCR genotyped to verify deletion of *ECE1* as described above.

The second set of *C*. *albicans* mutants was constructed in strain SC5314 using the transient CRISPR-Cas9 system [36] combined with the Nat flipper approach [38,39]. Briefly, the gRNA and Cas9 constructs were amplified from vector pV1093 (S3 Table) by PCR [40]. The gene deletion constructs with the maltose promoter-driven flippase and actin promoter-driven clonNAT selective marker were amplified with primers carrying micro-arms that were homologous to the flanking regions of target genes from a vector derived from pSF2A-mScarlet [38]. The gRNA, Cas9, and repair constructs were mixed and transformed into *C*. *albicans* cells using the lithium acetate heat-shock method [41]. Transformants were selected by growth on yeast extract peptone dextrose (YPD) agar containing with 200μg/mL of clonNAT. Successfull gene deletion was confirmed with multiple rounds of diagnostic PCRs, including confirming the integration of the deletion construct at the gene locus and the absence of the target gene, gRNA and Cas9 constructs. To recycle the dominant selective drug marker, confirmed transformants were cultured in YPM (YPD in which glucose was replaced with 2% maltose) broth for 2 days in a 30°C shaking incubator. 200–500 cells were then plated onto YPM plates. Individual colonies from YPM plates were then replicate plated onto YPM+clonNAT. NatS colonies were then picked and analyzed by PCR. Chromosomal rearrangement of constructed mutant strains was excluded by diagnostic PCR.

The plasmids containing wild-type *ECE1* and *ECE1*-V5 were constructed by fusion PCR. The P_*ACT1*_-*SAT1* fragments were amplified from vector pSFS2A-mScarlet with primers ACT1-F/ PolylinkerR and cloned into the EcoRI/HindIII sites of plasmid pUC19, generating vector pJL019. The P_*ECE1*_-*ECE1* fragments were amplified from SC5314 genomic DNA using primers ECE1_promoterF/ECE1_StopR and cloned into the EcoRI site of pJL019 to yield vector pJL034. Next, the terminator sequence of *ECE1* was amplified from SC5314 genomic DNA with primers ECE1_terF/ECE1_terR and cloned into the PstI/NotI sites of vector pJL034 to generate pJL044. The *ECE1*-V5 fragment was initially amplified by joint PCR to integrate a sequence containing the V5 epitope tag into the *ECE1* coding sequencing adjacent to the candidalysin Kex2 cleavage site as demonstrated in S6A Fig. The *ECE1*-V5 fragment was then cloned into pJL044 by replacing the sequences between EcoRI sites, generating vector pJL081. To complement the *ece1*Δ/Δ and *als3*Δ/Δ *ece1*Δ/Δ mutants, a fragment containing the *ECE1*, *ECE1*(-V5)-*SAT1* protein coding region, and *ECE1* terminator was amplified from plasmids pJL044 or pJL081 using primers ECEC-promoterF/ ECE1_terR and then transformed into the recipient strains. Heterozygous integration of the constructs at the native *ECE1* locus was confirmed by PCR.

For the experiments, the *C*. *albicans* cells were grown for 18 h in YPD broth in a shaking incubator at 30°C. The fungal cells were harvested by centrifugation, washed twice with phosphate-buffered saline (PBS), and counted using a hemacytometer.

### Dot blotting

The production of candidalysin-V5 by the various strains was detected by dot immunoblotting. *C*. *albicans* cells from YPD overnight culture were washed three times with PBS buffer and then inoculated into KSF medium (#37010022, Gibco,) at a concentration of 2x10^7^ cells/ml. After 7 hours of growing in a 37°C shaker, the culture supernatants were clarified twice by centrifugation at 3500*g* for 5 min. Culture supernatants were then passed through low protein binding 0.45μm PVDF filters to further remove any debris. The proteins in the supernatants were precipitated with 20% trichloracetic acid at 4°C overnight and collected by centrifugation at 15000*g* for 5 min. The pellets were washed twice with 100% ice-cold acetone and then dried at 90°C. For dot blotting, the dried proteins were suspended in SDS loading buffer and boiled for 10 min, after which serial 2-fold dilutions were spotted onto a 0.45μm PVDF transfer membrane for blotting. After blocking the membrane with 5% fat-free milk in TBST (0.05% Tween 20), it was incubated with mouse anti-V5 monoclonal antibody (#R960-25, ThermoFisher) in 5% fat-free milk in TBST, rinsed three times and then incubated with a HRP-labeled goat anti-mouse IgG secondary antibody-HRP (Invitrogen, #G21040). The spots were visualized by enhanced chemiluminescence (#AC2103, Azure Biosystems,).

### Oral epithelial cells

The OKF6/TERT-2 immortalized human oral epithelial cell line was kindly provided by J. Rheinwald (Harvard University, Cambridge, MA) [23] and was cultured as previously described [19]. OKF6/TERT-2 cells were authenticated by RNA-Seq [42] and tested for mycoplasma contamination. The TR146 buccal mucosa squamous cell carcinoma epithelial cell line was generously provided by J. R. Naglik (Kings College London, UK) and was cultured as previously described [14].

### Inhibitor and agonists

The EGFR kinase inhibitor gefitinib (Selleckchem) was dissolved in DMSO and used at a final concentration of 1 μm. It was added to the host cells 60 min prior to infection and remained in the medium for the entire incubation period. Control cells were incubated with a similar concentration of DMSO at a final concentration of 0.1%. EFNA1-Fc (Acro Biosystems) was used at a final concentration of 1 μg/ml, EGF was used at a concentration of 100 ng/ml and candidalysin was used at a concentration of 70 μM.

### siRNA

To knockdown EGFR and EphA2, OKF6/TERT-2 cells were transfected with siRNA as described previously [19]. Briefly, the cells were grown in 6-well tissue culture plates and transfected with 80 pmol EGFR siRNA (sc-29301, Santa Cruz Biotechnology), and EphA2 siRNA (sc-29304, Santa Cruz Biotechnology), or a similar amount of random control siRNA (sc-37007, Santa Cruz Biotechnology) using Lipofectamine 2000 (Thermo Fisher Scientific) following the manufacturer’s instructions. The extent of protein knockdown was verified 72 h later by immunoblotting with specific antibodies. Knockdown of the protein of interest was > 80%.

### Immunoblotting

OKF6/TERT-2 cells or TR146 cells in 24-well tissue culture plates were switched to supplement free KSF medium or DMEM/F12 medium, respectively for 1 h and then infected with 1 × 10^6^ *C*. *albicans* yeast for various times as described previously [10]. Next, the cells were rinsed with cold HBSS containing protease and phosphatase inhibitors, detached from the plate with a cell scraper, and collected by centrifugation. After boiling the cells in sample buffer, the resultant lysate was separated by SDS-PAGE, and phosphorylation was detected by immunoblotting with specific antibodies against pEphA2 (#6347, Cell Signaling) and pEGFR (#2234, Cell Signaling). Next, the blot was stripped, and the total amount of each protein was detected by immunoblotting with antibodies against EphA2 (D4A2, Cell Signaling), EGFR (#4267, Cell Signaling), and β-actin (#a5441, Sigma). Each experiment was performed at least 3 times.

### Immunoprecipitation

OKF6/TERT-2 cells were grown in 75 cm^2^ flasks to confluency, switched to KSF medium without supplements for 3 h, and then infected with 1x10^8^ *C*. *albicans* yeast. After 30 or 90 min. the cells were washed with ice-cold cold PBS (with Mg^2+^, and Ca^2+^), scraped from the flasks, and lysed with 100 μl ice-cold 5.8% octyl β-D-glucopyranoside (0479-5g; VWR) in the present of protease/phosphatase inhibitors. Whole cells lysates were precleared with 20μl of protein A/G plus (sc-2003; Santa Cruz Biotechnology) at 4°C for 30minutes. The bead-protein mix was centrifuged at 3000 rpm for 30 sec at 4°C and supernatants were collected. 2 μg of anti-EGFR antibody (sc-101; Santa Cruz Biotechnology) or anti-EphA2 antibody (#6347, Cell Signaling) was added to 500 μg of protein and incubated on a rotator at 4°C for 2 hours. 25μl of protein A/G plus was added to each immunoprecipitation sample and incubated for an additional hour at 4°C. Samples were pelleted at 3000 rpm for 30 sec, and washed 3 times in 500 μl of ice-cold 1.5% octyl β-D-glucopyranoside. Proteins were eluted with 30 μl of 2X SDS buffer and then heated at 90°C for 5 minutes. Samples were centrifuged at 3000 rpm for 30 sec after which the supernatants were collected, separated by SDS-PAGE, and analyzed as described above.

### Measurement of epithelial cell endocytosis

The endocytosis of *C*. *albicans* by oral epithelial cells was quantified as described previously [3]. OKF6/TERT-2 or TR146 oral epithelial cells were grown to confluency on fibronectin-coated circular glass coverslips in 24-well tissue culture plates and then infected for 120 min with 2×10^5^ yeast-phase *C*. *albicans* cells per well, after which they were fixed, stained, and mounted inverted on microscope slides. The coverslips were viewed with an epifluorescence microscope, and the number of endocytosed organisms per high-power field was determined, counting at least 100 organisms per coverslip. Each experiment was performed at least 3 times in triplicate.

### Immunofluorescence imaging

For the proximity ligation assays, OKF6/TERT-2 cells were seeded onto fibronectin treated coverslips in a 24 well plate overnight. The next day, the cells were infected with 3x10^5^ cells of yeast-phase *C*. *albicans* SC5314 for 90 min and then fixed with either 4% paraformaldehyde (for EphA2/EGFR) for 10 min at room temperature or 100% ice cold methanol for 20 min (for EphA2/HER2). After the cells were permeablized with 0.1% Triton X-100 in PBS for 15 min, they were incubated with a rabbit anti-EphA2 antibody (# 6997S, clone D4A2, Cell Signaling and either a mouse anti-EGFR antibody (# SC-101, clone R-1, Santa Cruz Biotechnology) or a mouse anti-HER2 antibody (# SC33684, clone 3B5, Santa Cruz Biotechnology). The interactions between EphA2 and either EGFR or HER2 were detected using the Duolink *In Situ* Red Starter Kit Mouse/Rabbit (DUO92101-1kit, Sigma-Aldrich) following the manufacturer’s instructions. The *C*. *albicans* cells were visualized by staining with a rabbit anti-*Candida* antibody (#B65411R, Meridian Life Science) labeled with Alexa Fluor 488 (#A20181, Thermo Fisher Scientific). The cells were imaged using a Leica TCS SP8 confocal microscope. Z-stacked images were obtained and overlaid on each other using the Leica image processing software.

To visualize the candidalysin-V5, OKF6/TERT-2 cells on fibronectin-coated glass coverslips were infected with 1x10^5^ of *C*. *albicans* cells. After incubation for 90 min, the medium was aspirated and the cells were fixed with 4% paraformaldehyde for 15 min at room temperature. The cells were washed twice with PBS and tthe epithelial cells were permeabilized and blocked with PBS containing 5% goat serum and 0.2% saponin. The candidalysin-V5 was detected by incubating the cells with a rabbit anti-V5 monoclonal antibody (#13202, Cell Signaling) followed by an Alexa 568 conjugated goat anti-rabbit secondary antibody (#A11031, Invitrogen). The *C*. *albicans* cells were stained with an Alexa 488-labeled anti-*Candida* antibody and the epithelial cell nuclei were stained with DAPI. The coverslips were mounted inverted in Gelvatol on microscopic slides and then imaged by confocal microscopy as described above.

### Cytokine and chemokine measurements *in vitro*

Cytokine levels in culture supernatants were determine as previously described [10]. Briefly OKF6/TERT-2 cells or TR146 cells in a 96-well plate were infected with *C*. *albicans* at a multiplicity of infection of 5. After 8 h of infection, the medium above the cells was collected, clarified by centrifugation and stored in aliquots at -80°C. The concentration of inflammatory cytokines and chemokines in the medium was determined using the Luminex multipex assay (R&D Systems).

### Epithelial cell damage

The effects of gefitinib on extent of epithelial cell damage caused by *C*. *albicans* was determined by our previously described ^51^Cr release assay [3]. OKF6/TERT-2 cells and TR146 cells in a 24-well plate were loaded with ^51^Cr overnight. The next day, they were incubated with gefitinib or diluent and then infected with *C*. *albicans* at a multiplicity of infection of 10. At various time points, the medium above the epithelial cells was collected and the epithelial cells were lysed with RadiacWash (Biodex). The amount of ^51^Cr released into the medium and remaining in the cells was determined with a gamma counter, and the percentage of ^51^Cr released in the infected cells we compared to the release by uninfected epithelial cells. The experiment was performed 3 times in triplicate.

### Mouse model of oropharyngeal candidiasis

Male, 6 week old BALB/c mice were purchased from Taconics and C57BL/6J mice were purchased from Jackson Laboratories. OPC was induced in mice as described previously [43]. Starting on day -2 relative to infection, the mice were randomly assigned to receive gefitinib or no treatment. Gefitinib was administered by adding the drug to the powdered chow diet at a final concentration of 200 parts-per-million. For inoculation, the animals were sedated, and a swab saturated with 2 × 10^7^ *C*. *albicans* cells was placed sublingually for 75 min. Mice were sacrificed after 1 and 2 days of infection. The tongues were harvested, weighed, homogenized and quantitatively cultured. The researchers were not blinded to the experimental groups because the endpoints (oral fungal burden, cytokine levels, and leukocyte numbers) were an objective measure of disease severity.

### Cytokine and chemokine measurements *in vivo*

To determine the whole tongue cytokine and chemokine protein concentrations, the mice were orally infected with *C*. *albicans* as above. After 1 and 2 days of infection, the mice were sacrificed, and their tongues were harvested, weighed and homogenized. The homogenates were cleared by centrifugation and the concentration of inflammatory mediators was measured using a multiplex bead array assay (R&D Systems) as previously described [10].

### Flow cytometry

To detect phosphorylation of EphA2, and EGFR in the tongue of *C*. *albicans* infected mice, the mice were orally infected with *C*. *albicans* as above. After 1 day of infection, the mice were sacrificed, and their tongues were harvested, fixed in 3% PFA for 30 min. After washing, a single cell suspension was prepared as described below. Single cells were permeabilized with methanol for 10 min, washed and stained with anti-pEphA2 (#6347, Cell Signaling) or anti-pEGFR (#2234, Cell Signaling) antibodies overnight at 4°C. The next day the cells were further stained with anti-rabbit FITC Ab (Abcam, ab6717), CD326 (Ep-CAM)-PE (G8.8, Biolegend), CD45-APC (30-F11; BD Biosciences) and then analyzed with a BD FACSymphony A5 flow cytometer.

The number of phagocytes in the mouse tongues were characterized as described previously [44,45]. Briefly, mice were orally infected with *C*. *albicans* as described above. After 1 d of infection, the animals were administered a sublethal anesthetic mix intraperitoneally. The thorax was opened, and a part of the rib cage removed to gain access to the heart. The vena cava was transected and the blood was flushed from the vasculature by slowly injecting 10 mL PBS into the right ventricle. The tongue was harvested and cut into small pieces in 100 μL of ice-cold PBS. 1 mL digestion mix (4.8 mg/ml Collagenase IV; Worthington Biochem, and 200 μg/ml DNase I; Roche Diagnostics, in 1x PBS) was added after which the tissue was incubated at 37°C for 45 min. The resulting tissue suspension was then passed through a 100 μm cell strainer. The single-cell suspensions were incubated with rat anti-mouse CD16/32 (2.4G2; BD Biosciences) for 10 min in FACS buffer at 4°C to block Fc receptors. For staining of surface antigens, cells were incubated with fluorochrome-conjugated (FITC, PE, PE-Cy7, allophycocyanin [APC], APC-eFluor 780,) antibodies against mouse CD45 (30-F11; BD Biosciences), Ly6C (AL-21; BD Biosciences), Ly6G (1A8, BioLegend), CD11b (M1/70; eBioscience), and CD90.2 (30-H12; BioLegend). After washing with FACS buffer, the cell suspension was stained with a LIVE/DEAD fluorescent dye (7-AAD; BD Biosciences) for 10 min. The stained cells were analyzed on a 2-laser LSRII flow cytometer (BD Biosciences), and the data were analyzed using FACS Diva (BD Biosciences) and FlowJo software (Treestar). Only single cells were analyzed, and cell numbers were quantified using PE-conjugated fluorescent counting beads (Spherotech).

### Phagocyte killing assays

The effects of gefitinib on neutrophil killing of *C*. *albicans* were determined by our previously described method [46]. To study human cells, neutrophils were isolated from the blood of healthy volunteers and incubated with gefitinib or diluent in RPMI 1640 medium plus 10% fetal bovine serum for 1 h at 37°C. Next, the neutrophils were mixed with an equal number of serum-opsonized *C*. *albicans* cells. After a 3 h incubation, the neutrophils were lysed by sonication, and the number of viable *C*. *albicans* cells was determined by quantitative culture.

To study bone marrow-derived neutrophils and macrophages (BMDMs), bone marrow cells from *BALB/c* mice (Taconics) were flushed from femurs and tibias using sterile RPMI 1640 medium supplemented with 10% fetal bovine serum (FBS) and 2 mM EDTA onto a 50 ml screw top Falcon tube fitted with a 100 μm filter [47]. Mouse neutrophils were purified from bone marrow cells using negative magnetic bead selection according to the manufacturer’s instructions (MojoSort, BioLegend). These neutrophils had > 90% purity and > 90% viability as determined by flow cytometry. To isolate BMDMs, 6x10^6^ bone marrow cells per 75 cm^2^ were seeded in RPMI 1640 supplemented with 20% FBS, 100 μg/ml streptomycin, 100 U/ml penicillin, 2 mM Glutamine, and 25 ng/ml rHu M-CSF (PeproTech). After 7 days, the BMDMs were treated with gefitinib or the diluent and then incubated with serum-opsonized *C*. *albicans* cells (multiplicity of infection 1:20) for 3 h. Next, the BMDMs were scraped, lysed by sonication, and the number of viable *C*. *albicans* cells was determined by quantitative culture.

### Statistics

At least three biological replicates were performed for all *in vitro* experiments unless otherwise indicated. Data were compared by analysis of variance with Dunnett’s test for multiple comparisons, the non-parametric Mann-Whitney, or the non-parametric one-way Kruskal-Wallis test followed by Dunn’s post-hoc test using GraphPad Prism (v. 8) software. P values < 0.05 were considered statistically significant.

## Supporting information

S1 Fig Interactions of EphA2 and EGFR. (A) Densitometric quantification of all 3 immunoblots such as the one in Fig 1A. (B) Effects of the EGFR kinase inhibitor gefitinib (GEF) on the time course of EphA2 phosphorylation in oral epithelial cells infected with *C*. *albicans*. Results are representative of 3 independent experiments. (C) Densitometric quantification of all 3 immunoblots such as the one in (B). Data in (A and C) were analyzed using the two-tailed Student’s t-test assuming unequal variances. **, *P* < 0.01.
(PDF)

S2 Fig Physical interactions of EphA2 with HER2 and EGFR in oral epithelial cells. (A) Proximity ligation assay to detect the interaction of EphA2 with HER2 in uninfected (Ctrl) oral epithelial cells and cells infected with *C*. *albicans* (Ca) for 90 min. (B) Lysates of oral epithelial cells infected with *C*. *albicans* for 30 and 90 min were immunoprecipitated (IP) with antibodies against EphA2 (left) and EGFR (right), after which EphA2 and EGFR were detected by immunoblotting (Top). Immunoblots of lysates prior to immunoprecipitation, demonstrating equal amounts of input protein (Bottom). Densitometric analysis of 3 independent immunoblots such as the ones shown in (B). Data were analyzed using the two-tailed Student’s t-test assuming unequal variances. NS, not significant.
(PDF)

S3 Fig Effect of receptor knockdown on cellular levels of phosphorylated and total EphA2 and EGFR. (A) Immunoblots showing effects of EphA2 (Left) or EGFR (Right) siRNA on total and phosphorylated EphA2 and EGFR in uninfected OKF6/TERT-2 oral epithelial cells. Results are representative of 3 independent experiments. (B) Densitometric quantification of all 3 immunoblots such as the one in S3A Fig. Data were analyzed using the two-tailed Student’s t-test assuming unequal variances. ****, *P* < 0.0001.
(PDF)

S4 Fig *C*. *albicans als3*Δ/Δ and *ece1*Δ/Δ mutant strains induce weak EGFR phosphorylation and transient phosphorylation of EphA2. Densitometric analysis of 3 EphA2 and EGFR phosphorylation (Y1068) (A) and total EphA2 and EGFR levels (B) in oral epithelial cells that had been infected with the indicated *C*. *albicans* strains for 30 and 90 min. Results are combined data from 3 immunoblots. Images of representative immunoblots are shown in Fig 2A. Data were analyzed using the two-tailed Student’s t-test assuming unequal variances. *, *P* < 0.05.
(PDF)

S5 Fig Effects of deletion of *ALS3* and/or *ECE1* on *C*. *albicans* adherence and invasion of oral epithelial cells. Data are the combined results of 3 experiments, each performed in triplicate. Orgs/HPF, organisms per high-power field; WT, wild-type; **, *P* < 0.01; ***, *P* < 0.001; ****, *P* <0.0001 by the Kruskal-Wallis test corrected for multiple comparisons.
(PDF)

S6 Fig Protein sequence of Candidalysin-V5. (A) Sequence alignment of wild-type Ece1 and Ece1-V5. Candidalysin is highlighted in green, Kex1/2 cutting sites are highlighted in yellow, and the V5 sequence is indicated by red font. (B) Scheme of candidalysin-V5. Basic and acidic amino acid are indicated.
(PDF)

S7 Fig Candidalysin-V5 is functional. (A) Secretion of candidalysin-V5 by the indicated *C*. *albicans* strain grown in KSF medium for 8 h at 37°C. as analyzed by dot immunoblotting with an anti-V5 antibody. (B) Epithelial cell damage caused by the indicated *C*. *albicans* strains after 8 h. (C) Control images showing absence of staining with the anti-V5 antibody in epithelial cells infected with *C*. *albicans* strains that do not contain *ECE1-V\5*. Insets show magnified views of the organisms.
(PDF)

S8 Fig Als3 and Ece1 are required for the phosphorylation of distinct EGFR tyrosine residues. (A) Densitometric analysis of 3 immunoblots to detect EGFR phosphorylation on the indicated tyrosine residues in oral epithelial cells that had been infected with the indicated *C*. *albicans* strains for 90 min. Images of representative immunoblots are shown in Fig 3A. Data were analyzed using the two-tailed Student’s t-test assuming unequal variances. *, *P* < 0.05. (C) Complementation of *als3*Δ/Δ and *ece1*Δ/Δ mutants restores EGFR phosphorylation. Results are representative of 2 independent experiments.
(PDF)

S9 Fig Gefitinib inhibits CXCL8/IL-8 and CCL20 in response to *C*. *albicans* infection. (A) Effects of the EGFR inhibitor gefitinib on the epithelial cell response to *C*. *albicans*. (B) Stimulation of epithelial cells by the indicated strains of *C*. *albicans*. Box whisker plots show median, interquartile range, and range of 3 independent experiments, each performed in duplicate. The data were analyzed using the Kruskal-Wallis test corrected for multiple comparisons. **, *P* < 0.01; ***, *P* < 0.001; ****, *P* < 0.0001; *Ca*, *C*. *albicans;* ctrl, control; GEF, gefitinib.
(PDF)

S10 Fig Densitometric analysis of EphA2 and EGFR phosphorylation and protein levels. (A and B). Phosphorylation of EphA2 in uninfected oral epithelial cells (UNINF) and epithelial cells exposed to ephrin A1-Fc (EFNA1-Fc) or yeast-phase *C*. *albicans* SC5314 (Ca) for 15 min (A) and 60 min (B). Left panel in (B) shows the levels of total EphA2 relative to β-actin at 60 min. (C) Effects of epidermal growth factor (EGF) on the phosphorylation and total levels of EphA2. (D) Effects of EFNA1 and EGF on the phosphorylation and total cellular levels of EphA2 and EGFR. (E and F) Phosphorylation and total levels of EphA2 (E) and EGFR (F) in oral epithelial cells exposed to candidalysin for 30 min and 90 min. Data are the mean ± SD of 3 independent immunoblots. Images of representative immunoblots are show in Fig 4F. Data were analyzed using the two-tailed Student’s t-test assuming unequal variances.*, *P* < 0.05, **, *P* < 0.01; ***, *P* < 0.001; ****, *P* < 0.0001.
(PDF)

S11 Fig Densitometric analysis of EphA2 and EGFR phosphorylation and protein levels. (A) Phosphorylation of EGFR in uninfected OKF6/TERT-2 and TR146 cells. (B) Phosphorylation of EphA2 and EGFR in TR146 cells exposed to yeast-phase *C*. *albicans* for 30 and 90 min. Data are the mean ± SD of 3 independent immunoblots. Images of representative immunoblots are shown in Fig 5E and 5F. Data were analyzed using the two-tailed Student’s t-test assuming unequal variances.*, *P* < 0.05, ***, *P* < 0.001.
(PDF)

S12 Fig Pharmacological inhibition of EGFR reduces *C*. *albicans*-induced EphA2 activation during OPC. (A) Gating strategy to determine EGFR and EphA2 phosphorylation in the oral epithelial cells of mice with OPC. (B) Representative histograms of CD45- EpCam^+^ cells showing the effects of *C*. *albicans* infection and gefitinib (GEF) treatment on the phosphorylation of EGFR and EphA2 after 1 d of OPC. (C and D) Effects of gefitinib on the percentage of oral epithelial cells with phosphorylated EGFR (C) and EphA2 (D) in mice after 1 d of OPC. Data are combined results from 6 mice per group from a single experiment. Statistical significance was determined using the Mann-Whitney test. *, *p* < 0.05; **, *p* < 0.01; INF, infected; UNINF, uninfected. (E) Gating strategies used to identify Ly6C^hi^ inflammatory monocytes and Ly6C^+^ neutrophils in the flow cytometric analysis of the tongue digests. The results from these experiments are shown in Fig 6C and 6D.
(PDF)

S13 Fig Gefitinib has no effect on the killing of *C*. *albicans* by phagocytes. (A-C) The percentage of *C*. *albicans* cells killed by human neutrophils exposed to gefitinib *ex vivo* (A), bone marrow neutrophils isolated from gefitinib treated mice (B), and mouse bone marrow derived macrophages (BMDM) treated with gefitinib *ex vivo* (C). Data are the combined results of 3 experiments, each performed in duplicate. Data were analyzed using the two-tailed Student’s t-test assuming unequal variances. NS, not significant.
(PDF)

S14 Fig Deletion of *ECE1* results in lower oral fungal burden. Oral fungal burden of immunocompetent C57BL/6 mice infected with indicated strains of *C*. *albicans* after 2 days of infection. Results are median of a total of 5 mice per group from a single experiment. The y-axis is set at the limit of detection (20 CFU/g tissue). Data were analyzed using the Mann-Whitney test.*, *p* < 0.05.
(PDF)

S15 Fig Epithelial interactions of the indicated *C*. *albicans* strains. Data are the combined results of three experiments, each performed in triplicate. Statistical significance was determined by the Brown-Forsythe and Welch ANOVA test. NS, not significant; orgs/HPF, organisms per high power field; WT, wild-type; *, *p* <0.05; *p* < 0.01; ***, *p* < 0.001; ****,*p* < 0.0001.
(PDF)

S1 Table List of *C*. *albicans* strains used in the experiments. (PDF)

S2 Table List of primers used in the experiments. (PDF)

S3 Table List of plasmids. (PDF)
