# Supplementary material for: Activation of EphA2-EGFR signaling in oral epithelial cells by Candida albicans virulence factors
Source: PLoS Pathog. 2021 Jan 20;17(1):e1009221. doi: 10.1371/journal.ppat.1009221 (PMC7850503; doi:10.1371/journal.ppat.1009221)
Supplement: S1 Fig — (A) Densitometric quantification of all 3 immunoblots such as the one in Fig 1A. (B) Effects of the EGFR kinase inhibitor gefitinib (GEF) on the time course of EphA2 phosphorylation in oral epithelial cells infected with C. albicans. Results are representative of 3 independent experiments. (C) Densitometric quantification of all 3 immunoblots such as the one in (B). Data in (A and C) were analyzed using the two-tailed Student’s t-test assuming unequal variances. **, P < 0.01. (PDF) [file ppat.1009221.s001.pdf]

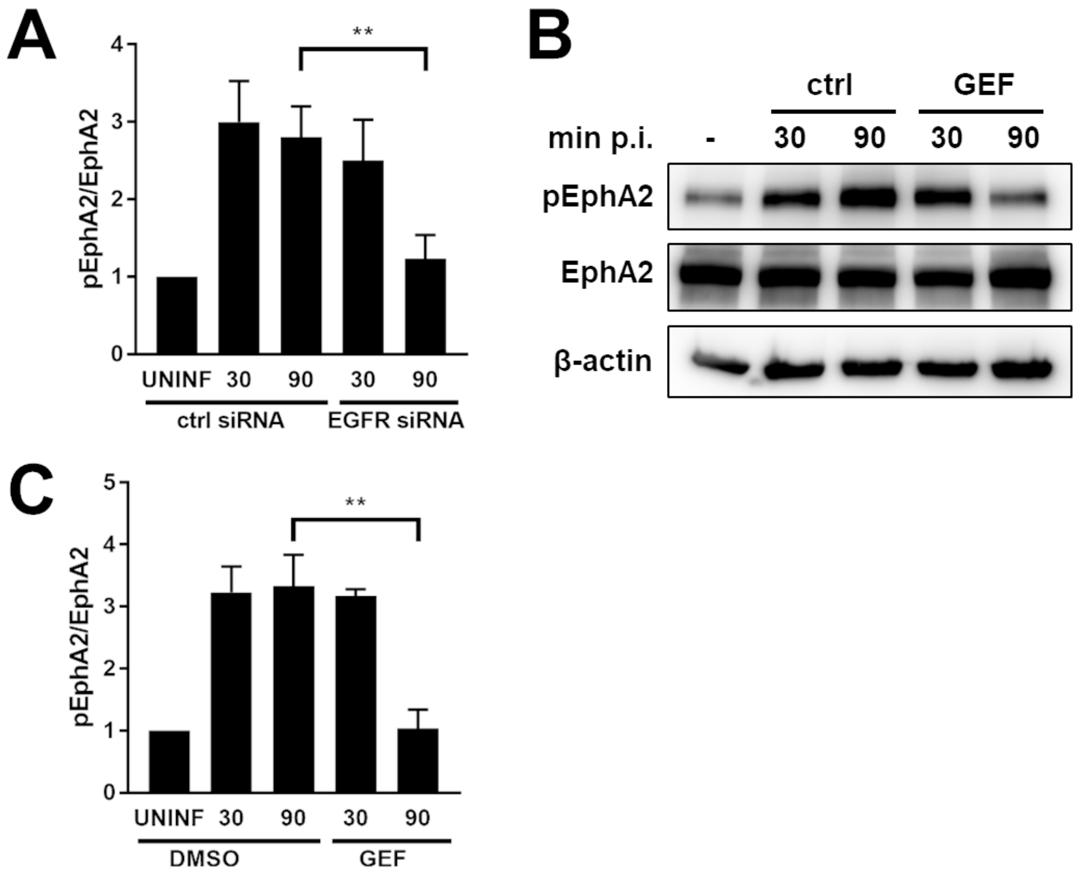

S1 Fig. Interactions of EphA2 and EGFR. (A) Densitometric quantification of all 3 immunoblots such as the one in Fig 1A. (B) Effects of EGFR kinase inhibitor gefitinib (GEF) on the time course of EphA2 phosphorylation in oral epithelial cells infected with *C. albicans*. Results are representative of 3 independent experiments. (C) Densitometric quantification of all 3 immunoblots such as the one in (B). Data in (A and C) were analyzed using the two-tailed Student's t-test assuming unequal variances. **, $P < 0.01$.
