# Supplementary material for: Activation of EphA2-EGFR signaling in oral epithelial cells by Candida albicans virulence factors
Source: PLoS Pathog. 2021 Jan 20;17(1):e1009221. doi: 10.1371/journal.ppat.1009221 (PMC7850503; doi:10.1371/journal.ppat.1009221)
Supplement: S2 Fig — (A) Proximity ligation assay to detect the interaction of EphA2 with HER2 in uninfected (Ctrl) oral epithelial cells and cells infected with C. albicans (Ca) for 90 min. (B) Lysates of oral epithelial cells infected with C. albicans for 30 and 90 min were immunoprecipitated (IP) with antibodies against EphA2 (left) and EGFR (right), after which EphA2 and EGFR were detected by immunoblotting (Top). Immunoblots of lysates prior to immunoprecipitation, demonstrating equal amounts of input protein (Bottom). Densitometric analysis of 3 independent immunoblots such as the ones shown in (B). Data were analyzed using the two-tailed Student’s t-test assuming unequal variances. NS, not significant. (PDF) [file ppat.1009221.s002.pdf]

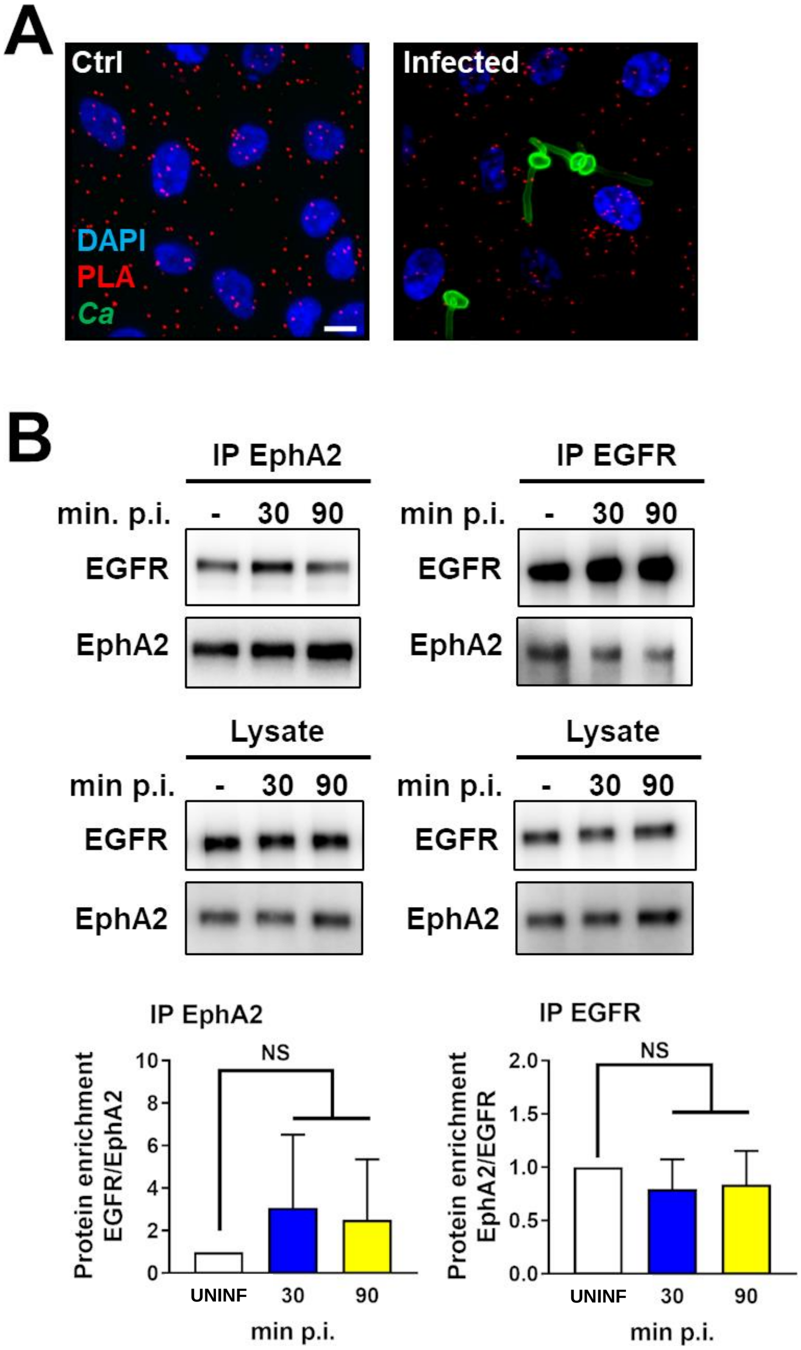

S2 Fig. Physical interactions of EphA2 with HER2 and EGFR in oral epithelial cells, (A) Proximity ligation assay to detect the interaction of EphA2 with HER2 in uninfected (Ctrl) oral epithelial cells and cells infected with *C. albicans* (Ca) for 90 min. (B) Lysates of oral epithelial cells infected with *C. albicans* for 30 and 90 min were immunoprecipitated (IP) with antibodies against EphA2 (left) and EGFR (right), after which EphA2 and EGFR were detected by immunoblotting (Top). Immunoblots of lysates prior to immunoprecipitation, demonstrating equal amounts of input protein (Bottom). Densitometric analysis of 3 independent immunoblots such as the ones shown in (B). Data were analyzed using the two-tailed Student's t-test assuming unequal variances. NS, not significant.
