# Supplementary material for: Activation of EphA2-EGFR signaling in oral epithelial cells by Candida albicans virulence factors
Source: PLoS Pathog. 2021 Jan 20;17(1):e1009221. doi: 10.1371/journal.ppat.1009221 (PMC7850503; doi:10.1371/journal.ppat.1009221)
Supplement: S3 Fig — (A) Immunoblots showing effects of EphA2 (Left) or EGFR (Right) siRNA on total and phosphorylated EphA2 and EGFR in uninfected OKF6/TERT-2 oral epithelial cells. Results are representative of 3 independent experiments. (B) Densitometric quantification of all 3 immunoblots such as the one in S3A Fig. Data were analyzed using the two-tailed Student’s t-test assuming unequal variances. ****, P < 0.0001. (PDF) [file ppat.1009221.s003.pdf]

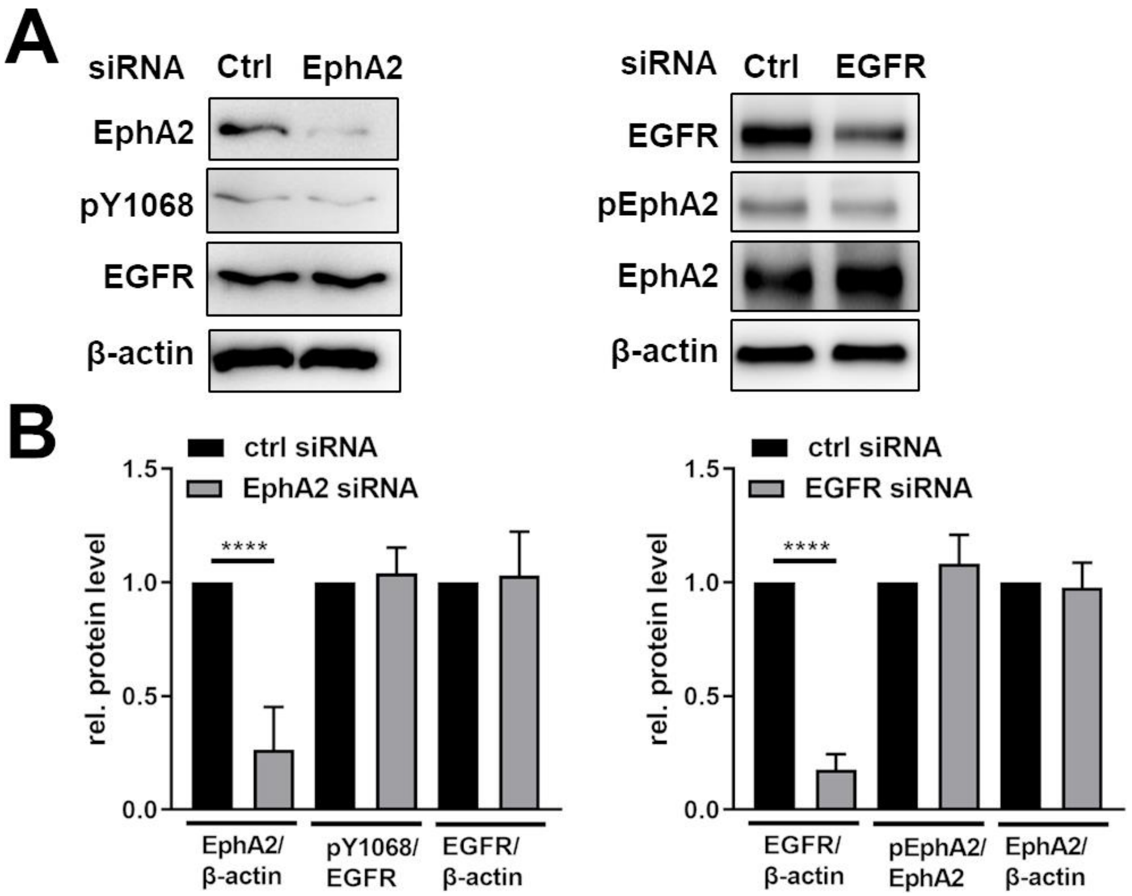

S3 Fig. Effect of receptor knockdown on cellular levels of phosphorylated and total EphA2 and EGFR. (A) Immunoblots showing effects of EphA2 (Left) or EGFR (Right) siRNA on total and phosphorylated EphA2 and EGFR in uninfected OKF6/TERT-2 oral epithelial cells. Results are representative of 3 independent experiments. (B) Densitometric quantification of all 3 immunoblots such as the one in Fig (A). Data were analyzed using the two-tailed Student's t-test assuming unequal variances. ****, $P < 0.0001$.
