# Supplementary material for: Activation of EphA2-EGFR signaling in oral epithelial cells by Candida albicans virulence factors
Source: PLoS Pathog. 2021 Jan 20;17(1):e1009221. doi: 10.1371/journal.ppat.1009221 (PMC7850503; doi:10.1371/journal.ppat.1009221)
Supplement: S4 Fig — Densitometric analysis of 3 EphA2 and EGFR phosphorylation (Y1068) (A) and total EphA2 and EGFR levels (B) in oral epithelial cells that had been infected with the indicated C. albicans strains for 30 and 90 min. Results are combined data from 3 immunoblots. Images of representative immunoblots are shown in Fig 2A. Data were analyzed using the two-tailed Student’s t-test assuming unequal variances. *, P < 0.05. (PDF) [file ppat.1009221.s004.pdf]

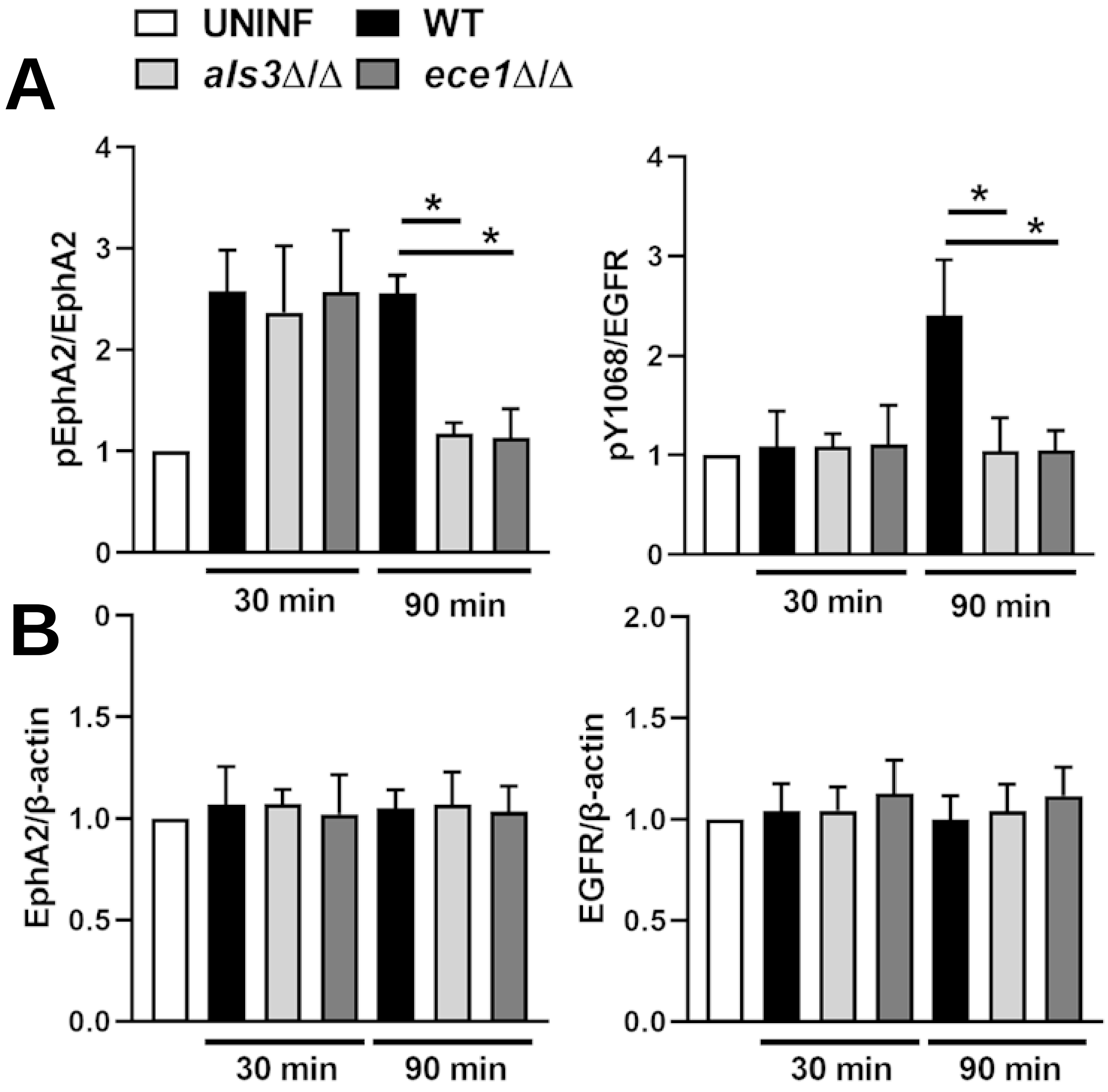

S4 Fig. *C. albicans als3*Δ/Δ and *ece1*Δ/Δ mutant strains induce weak EGFR phosphorylation and transient phosphorylation of EphA2. Densitometric analysis of 3 EphA2 and EGFR phosphorylation (Y1068) (A) and total EphA2 and EGFR levels (B) in oral epithelial cells that had been infected with the indicated *C. albicans* strains for 30 and 90 min. Results are combined data from 3 immunoblots. Images of representative immunoblots are shown in Fig 2A. Data were analyzed using the two-tailed Student's t-test assuming unequal variances. *, $P < 0.05$.
