# Supplementary material for: Activation of EphA2-EGFR signaling in oral epithelial cells by Candida albicans virulence factors
Source: PLoS Pathog. 2021 Jan 20;17(1):e1009221. doi: 10.1371/journal.ppat.1009221 (PMC7850503; doi:10.1371/journal.ppat.1009221)
Supplement: S5 Fig — Data are the combined results of 3 experiments, each performed in triplicate. Orgs/HPF, organisms per high-power field; WT, wild-type; **, P < 0.01; ***, P < 0.001; ****, P <0.0001 by the Kruskal-Wallis test corrected for multiple comparisons. (PDF) [file ppat.1009221.s005.pdf]

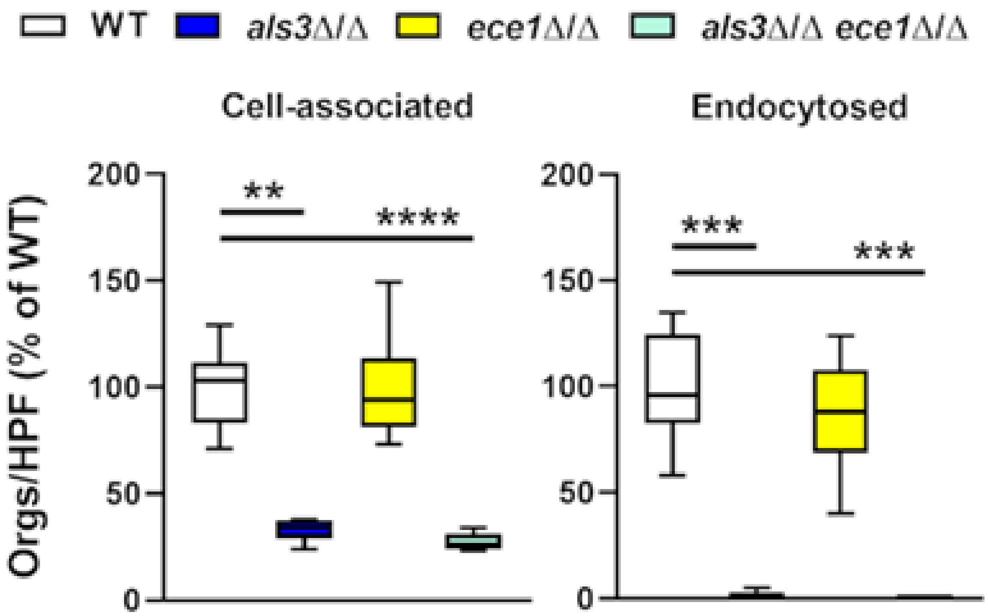

S5 Fig. Effects of deletion of *ALS3* and/or *ECE1* on *C. albicans* adherence and invasion of oral epithelial cells. Data are the combined results of 3 experiments, each performed in triplicate. Orgs/HPF, organisms per high-power field; WT, wild-type; **, *P* < 0.01; ***, *P* < 0.001; ****, *P* <0.0001 by the Kruskal-Wallis test corrected for multiple comparisons.
