# Supplementary material for: Activation of EphA2-EGFR signaling in oral epithelial cells by Candida albicans virulence factors
Source: PLoS Pathog. 2021 Jan 20;17(1):e1009221. doi: 10.1371/journal.ppat.1009221 (PMC7850503; doi:10.1371/journal.ppat.1009221)
Supplement: S6 Fig — (A) Sequence alignment of wild-type Ece1 and Ece1-V5. Candidalysin is highlighted in green, Kex1/2 cutting sites are highlighted in yellow, and the V5 sequence is indicated by red font. (B) Scheme of candidalysin-V5. Basic and acidic amino acid are indicated. (PDF) [file ppat.1009221.s006.pdf]

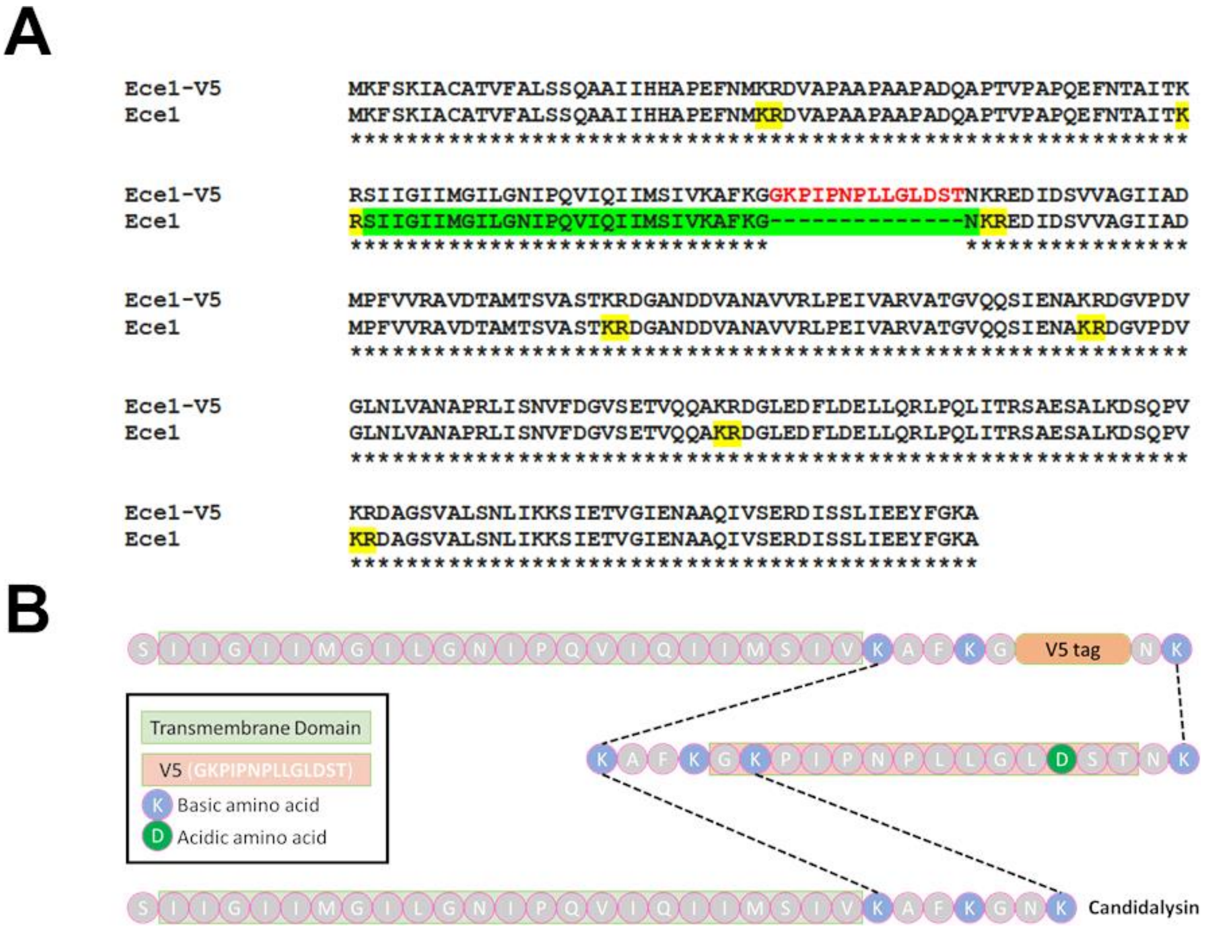

S6 Fig. Protein sequence of Candidalysin-V5. (A) Sequence alignment of wild-type Ece1 and Ece1-V5. Candidalysin is highlighted in green, Kex1/2 cutting sites are highlighted in yellow, and the V5 sequence is indicated by red font. (B) Scheme of Candidalysin-V5. Basic and acidic amino acid are indicated.
