# Supplementary material for: Activation of EphA2-EGFR signaling in oral epithelial cells by Candida albicans virulence factors
Source: PLoS Pathog. 2021 Jan 20;17(1):e1009221. doi: 10.1371/journal.ppat.1009221 (PMC7850503; doi:10.1371/journal.ppat.1009221)
Supplement: S7 Fig — (A) Secretion of candidalysin-V5 by the indicated C. albicans strain grown in KSF medium for 8 h at 37°C. as analyzed by dot immunoblotting with an anti-V5 antibody. (B) Epithelial cell damage caused by the indicated C. albicans strains after 8 h. (C) Control images showing absence of staining with the anti-V5 antibody in epithelial cells infected with C. albicans strains that do not contain ECE1-V\5. Insets show magnified views of the organisms. (PDF) [file ppat.1009221.s007.pdf]

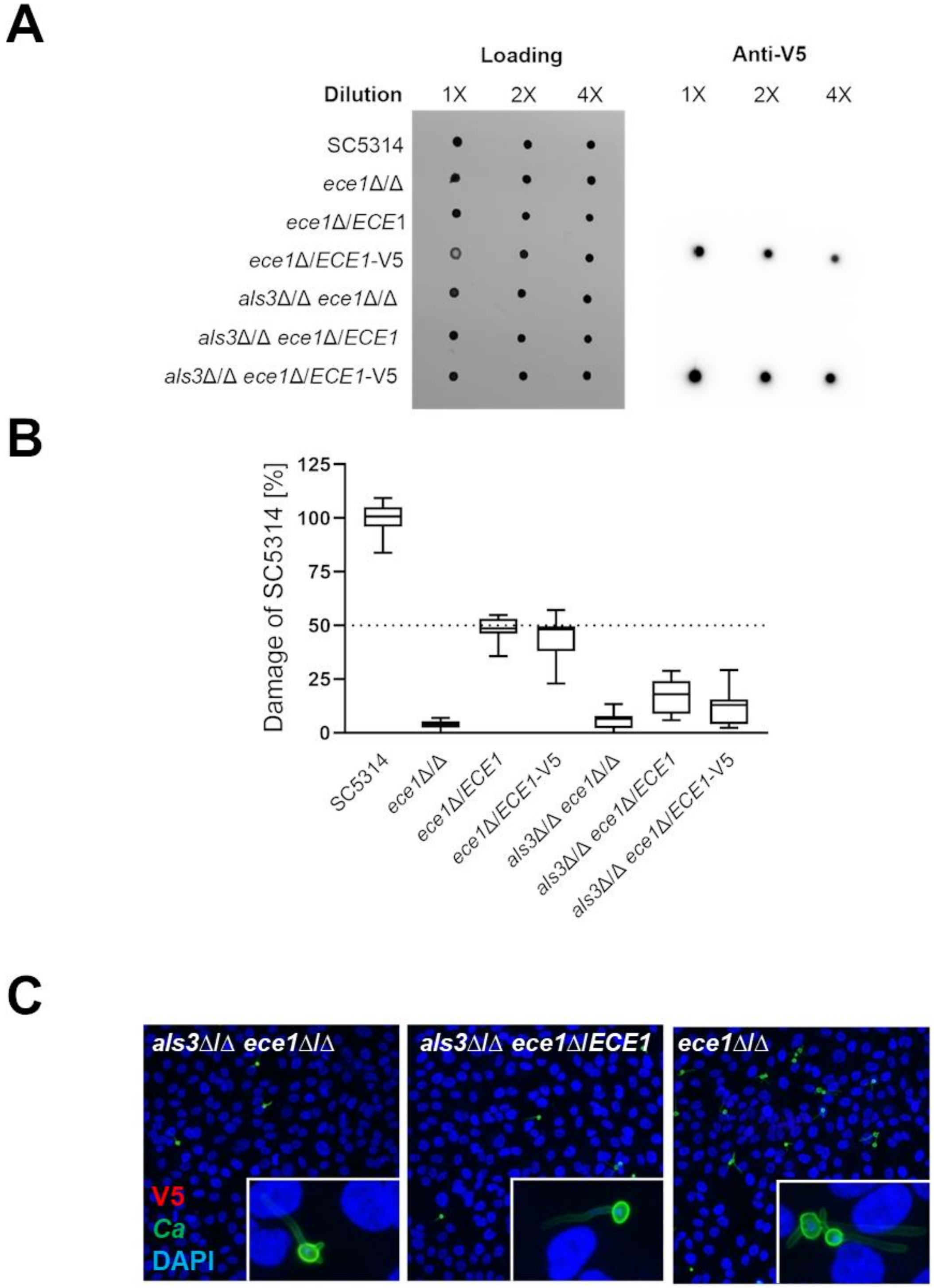

S7 Fig. Candidalysin-V5 is functional. (A) Secretion of Candidalysin-V5 by the indicated *C. albicans* strain grown in KSF medium for 8 h at 37°C. as analyzed by dot immunoblotting with an anti-V5 antibody. (B) Epithelial cell damage caused by the indicated *C. albicans* strains after 8 h. (C) Control images showing absence of staining with the anti-V5 antibody in epithelial cells infected with *C. albicans* strains that do not contain *ECE1-V\5*. Insets show magnified views of the organisms.
