# Supplementary material for: Activation of EphA2-EGFR signaling in oral epithelial cells by Candida albicans virulence factors
Source: PLoS Pathog. 2021 Jan 20;17(1):e1009221. doi: 10.1371/journal.ppat.1009221 (PMC7850503; doi:10.1371/journal.ppat.1009221)
Supplement: S8 Fig — (A) Densitometric analysis of 3 immunoblots to detect EGFR phosphorylation on the indicated tyrosine residues in oral epithelial cells that had been infected with the indicated C. albicans strains for 90 min. Images of representative immunoblots are shown in Fig 3A. Data were analyzed using the two-tailed Student’s t-test assuming unequal variances. *, P < 0.05. (C) Complementation of als3Δ/Δ and ece1Δ/Δ mutants restores EGFR phosphorylation. Results are representative of 2 independent experiments. (PDF) [file ppat.1009221.s008.pdf]

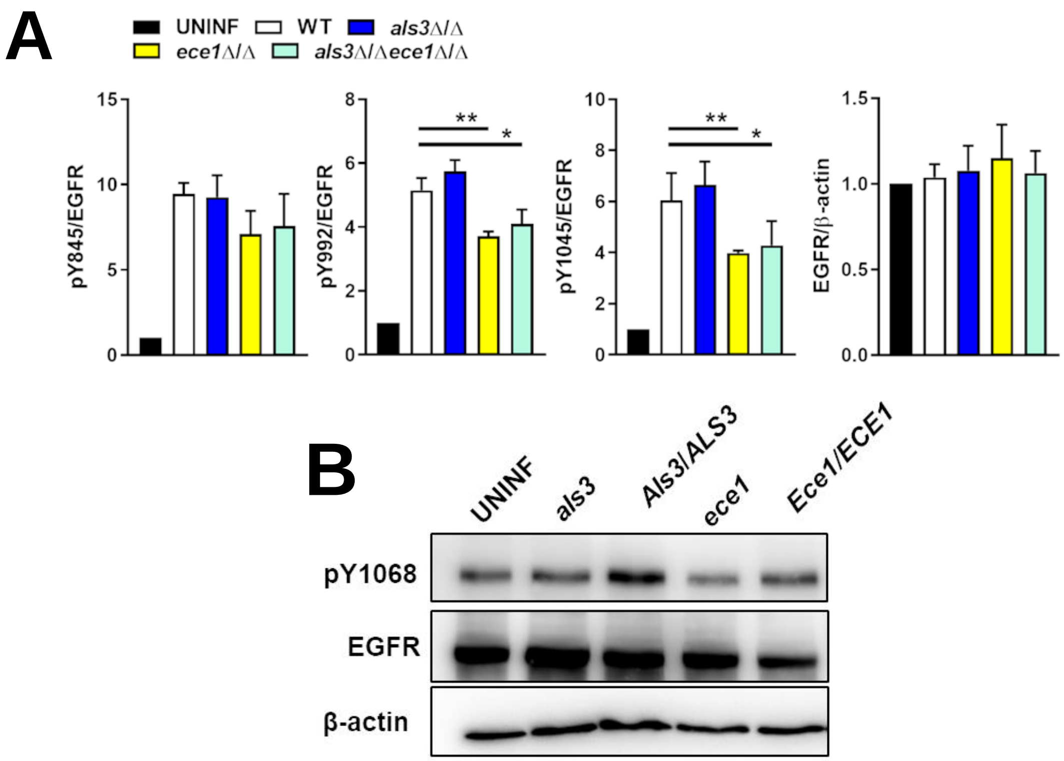

S8 Fig. Als3 and Ece1 are required for the phosphorylation of distinct EGFR tyrosine residues. (A) Densitometric analysis of 3 immunoblots to detect EGFR phosphorylation on the indicated tyrosine residues in oral epithelial cells that had been infected with the indicated *C. albicans* strains for 90 min. Images of representative immunoblots are shown in Fig 3A. Data were analyzed using the two-tailed Student's t-test assuming unequal variances. *, $P < 0.05$. (C) Complementation of *als3*Δ/Δ and *ece1*Δ/Δ mutants restores EGFR phosphorylation. Results are representative of 2 independent experiments.
