# Supplementary material for: Activation of EphA2-EGFR signaling in oral epithelial cells by Candida albicans virulence factors
Source: PLoS Pathog. 2021 Jan 20;17(1):e1009221. doi: 10.1371/journal.ppat.1009221 (PMC7850503; doi:10.1371/journal.ppat.1009221)
Supplement: S9 Fig — (A) Effects of the EGFR inhibitor gefitinib on the epithelial cell response to C. albicans. (B) Stimulation of epithelial cells by the indicated strains of C. albicans. Box whisker plots show median, interquartile range, and range of 3 independent experiments, each performed in duplicate. The data were analyzed using the Kruskal-Wallis test corrected for multiple comparisons. **, P < 0.01; ***, P < 0.001; ****, P < 0.0001; Ca, C. albicans; ctrl, control; GEF, gefitinib. (PDF) [file ppat.1009221.s009.pdf]

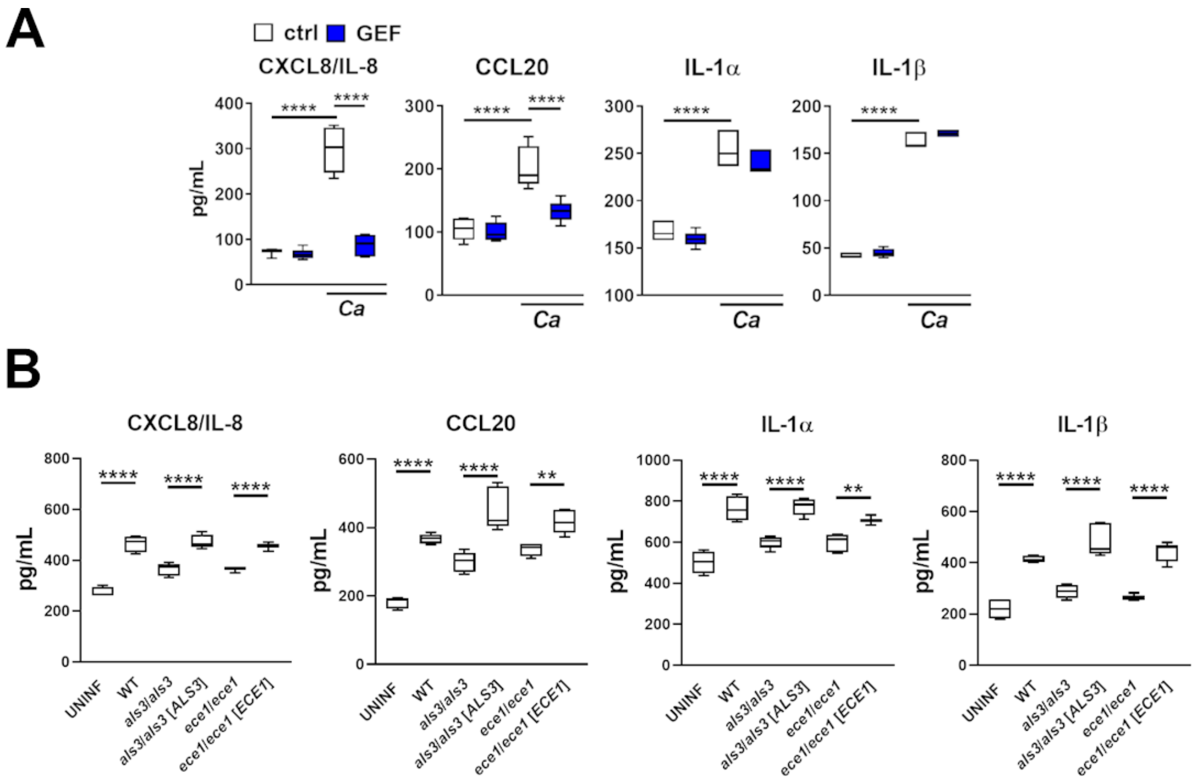

S9 Fig. Gefitinib inhibits CXCL8/IL-8 and CCL20 in response to *C. albicans* infection. (A) Effects of the EGFR inhibitor gefitinib on the epithelial cell response to *C. albicans*. (B) Stimulation of epithelial cells by the indicated strains of *C. albicans.* Box whisker plots show median, interquartile range, and range of 3 independent experiments, each performed in duplicate. The data were analyzed using the Kruskal-Wallis test corrected for multiple comparisons. **, $P < 0.01$; ***, $P < 0.001$; ****, $P < 0.0001$; *Ca*, *C. albicans;* ctrl, control; GEF, gefitinib.
