# Supplementary material for: Activation of EphA2-EGFR signaling in oral epithelial cells by Candida albicans virulence factors
Source: PLoS Pathog. 2021 Jan 20;17(1):e1009221. doi: 10.1371/journal.ppat.1009221 (PMC7850503; doi:10.1371/journal.ppat.1009221)
Supplement: S10 Fig — (A and B). Phosphorylation of EphA2 in uninfected oral epithelial cells (UNINF) and epithelial cells exposed to ephrin A1-Fc (EFNA1-Fc) or yeast-phase C. albicans SC5314 (Ca) for 15 min (A) and 60 min (B). Left panel in (B) shows the levels of total EphA2 relative to β-actin at 60 min. (C) Effects of epidermal growth factor (EGF) on the phosphorylation and total levels of EphA2. (D) Effects of EFNA1 and EGF on the phosphorylation and total cellular levels of EphA2 and EGFR. (E and F) Phosphorylation and total levels of EphA2 (E) and EGFR (F) in oral epithelial cells exposed to candidalysin for 30 min and 90 min. Data are the mean ± SD of 3 independent immunoblots. Images of representative immunoblots are show in Fig 4F. Data were analyzed using the two-tailed Student’s t-test assuming unequal variances.*, P < 0.05, **, P < 0.01; ***, P < 0.001; ****, P < 0.0001. (PDF) [file ppat.1009221.s010.pdf]

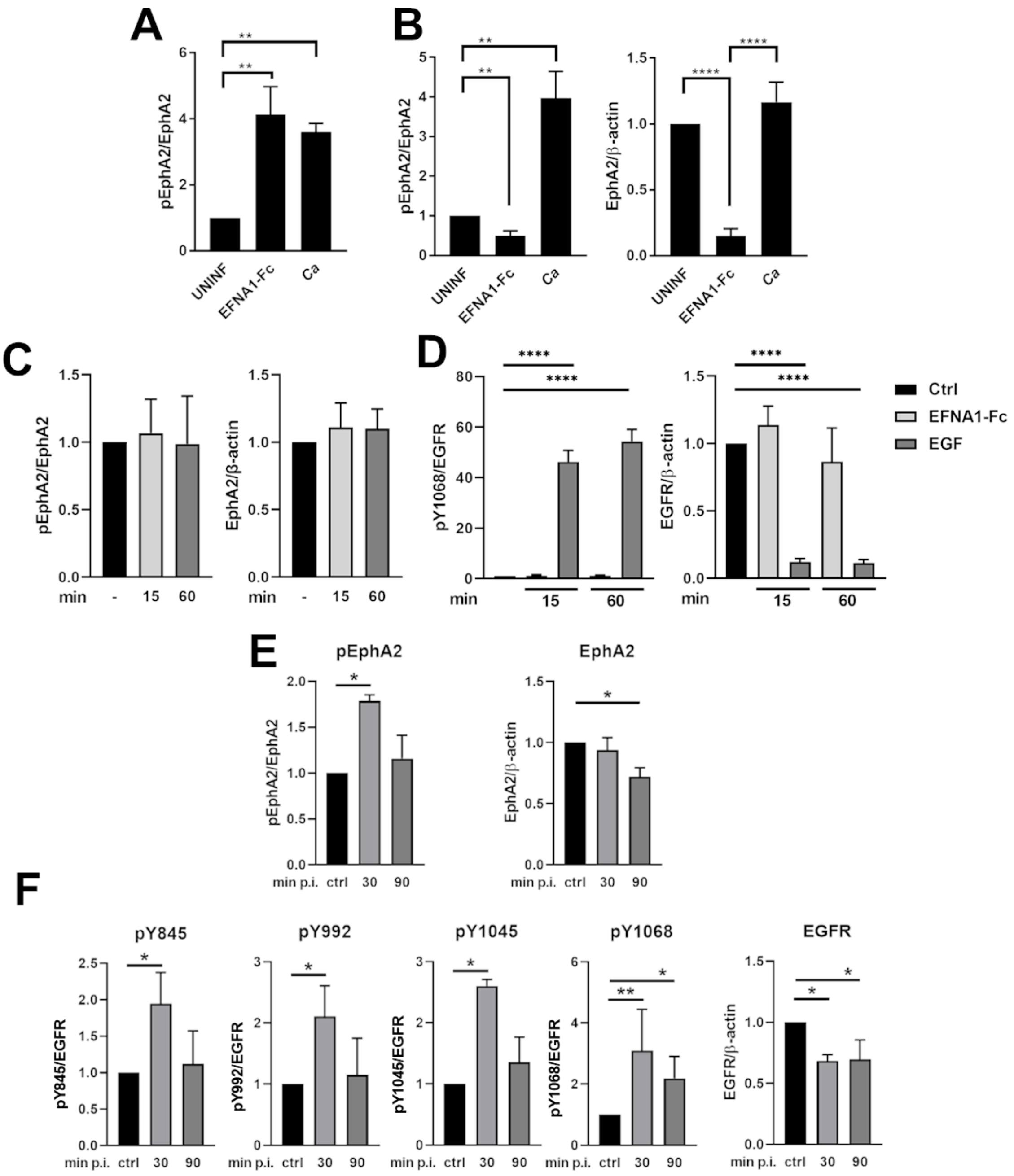

S10 Fig. Densitometric analysis of EphA2 and EGFR phosphorylation and protein levels. (A and B). Phosphorylation of EphA2 in uninfected oral epithelial cells (UNINF) and epithelial cells exposed to ephrin A1-Fc (EFNA1-Fc) or yeast-phase *C. albicans* SC5314 (Ca) for 15 min (A) and 60 min (B). Left panel in (B) shows the levels of total EphA2 relative to β-actin at 60 min. (C) Effects of epidermal growth factor (EGF) on the phosphorylation and total levels of EphA2. (D) Effects of EFNA1 and EGF on the phosphorylation and total cellular levels of EphA2 and EGFR. (E and F) Phosphorylation and total levels of EphA2 (E) and EGFR (F) in oral epithelial cells exposed to Candidalysin for 30 min and 90 min. Data are the mean ± SD of 3 independent immunoblots. Images of representative immunoblots are show in Fig 4-F. Data were analyzed using the two-tailed Student's t-test assuming unequal variances.*, $P < 0.05$, **, $P < 0.01$; ***, $P < 0.001$; ****, $P < 0.0001$.
