# Supplementary material for: Activation of EphA2-EGFR signaling in oral epithelial cells by Candida albicans virulence factors
Source: PLoS Pathog. 2021 Jan 20;17(1):e1009221. doi: 10.1371/journal.ppat.1009221 (PMC7850503; doi:10.1371/journal.ppat.1009221)
Supplement: S11 Fig — (A) Phosphorylation of EGFR in uninfected OKF6/TERT-2 and TR146 cells. (B) Phosphorylation of EphA2 and EGFR in TR146 cells exposed to yeast-phase C. albicans for 30 and 90 min. Data are the mean ± SD of 3 independent immunoblots. Images of representative immunoblots are shown in Fig 5E and 5F. Data were analyzed using the two-tailed Student’s t-test assuming unequal variances.*, P < 0.05, ***, P < 0.001. (PDF) [file ppat.1009221.s011.pdf]

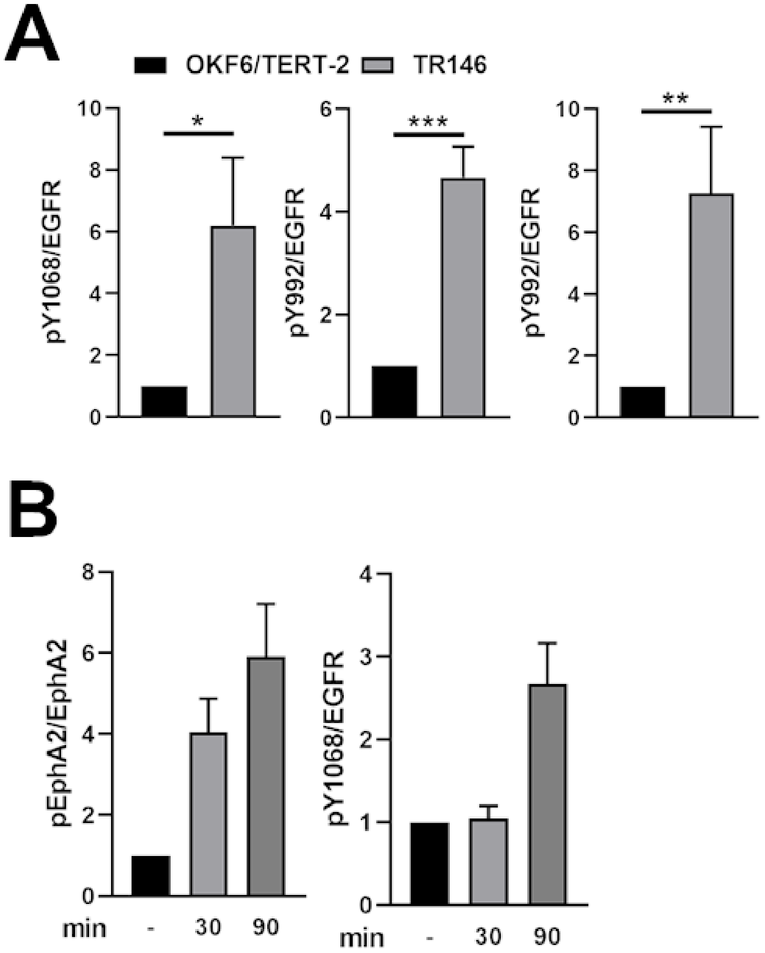

S11 Fig. Densitometric analysis of EphA2 and EGFR phosphorylation and protein levels. (A) Phosphorylation of EGFR in uninfected OKF6/TERT-2 and TR146 cells. (B) Phosphorylation of EphA2 and EGFR in TR146 cells exposed to yeast-phase *C. albicans* for 30 and 90 min. Data are the mean ± SD of 3 independent immunoblots. Images of representative immunoblots are show in Fig 5E and 5F. Data were analyzed using the two-tailed Student's t-test assuming unequal variances.*, $P < 0.05$, ***, $P < 0.001$.
