# Supplementary material for: Activation of EphA2-EGFR signaling in oral epithelial cells by Candida albicans virulence factors
Source: PLoS Pathog. 2021 Jan 20;17(1):e1009221. doi: 10.1371/journal.ppat.1009221 (PMC7850503; doi:10.1371/journal.ppat.1009221)
Supplement: S12 Fig — (A) Gating strategy to determine EGFR and EphA2 phosphorylation in the oral epithelial cells of mice with OPC. (B) Representative histograms of CD45- EpCam+ cells showing the effects of C. albicans infection and gefitinib (GEF) treatment on the phosphorylation of EGFR and EphA2 after 1 d of OPC. (C and D) Effects of gefitinib on the percentage of oral epithelial cells with phosphorylated EGFR (C) and EphA2 (D) in mice after 1 d of OPC. Data are combined results from 6 mice per group from a single experiment. Statistical significance was determined using the Mann-Whitney test. *, p < 0.05; **, p < 0.01; INF, infected; UNINF, uninfected. (E) Gating strategies used to identify Ly6Chi inflammatory monocytes and Ly6C+ neutrophils in the flow cytometric analysis of the tongue digests. The results from these experiments are shown in Fig 6C and 6D. (PDF) [file ppat.1009221.s012.pdf]

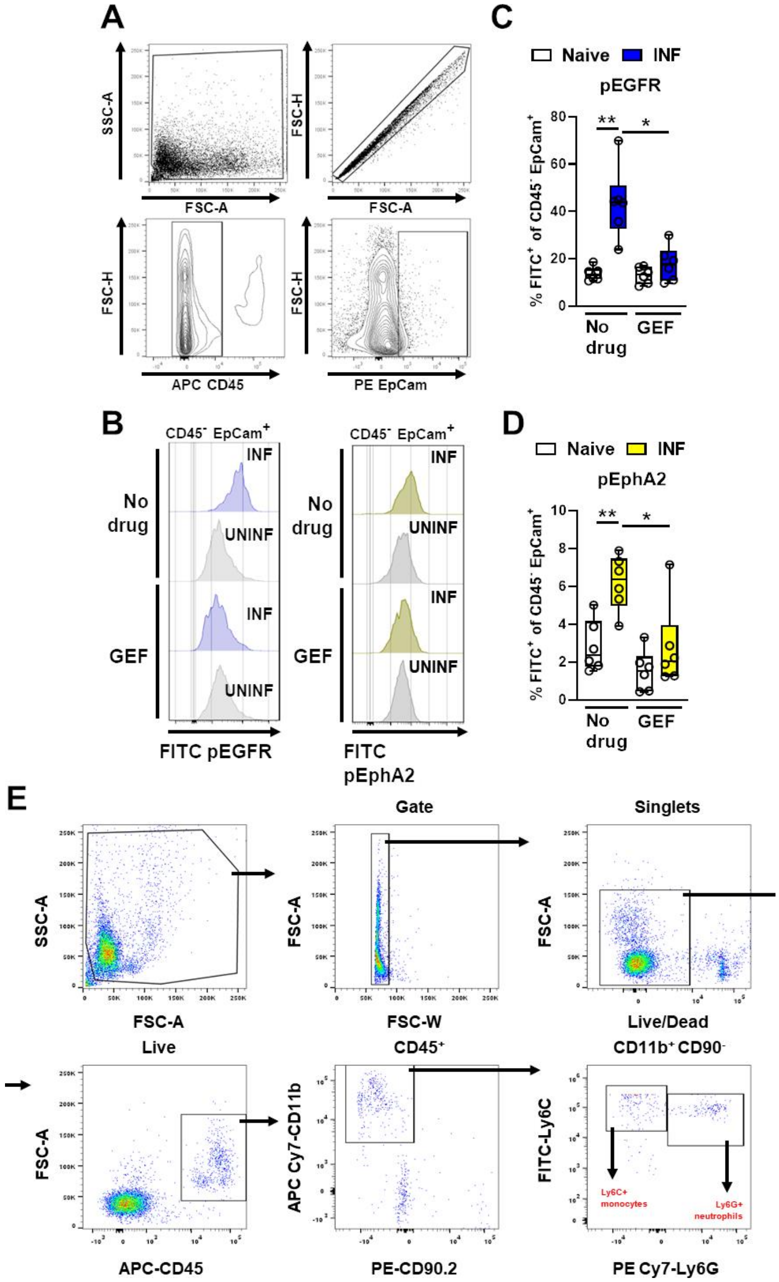

S12 Fig. Pharmacological inhibition of EGFR reduces *C. albicans*-induced EphA2 activation during OPC. (A) Gating strategy to determine EGFR and EphA2 phosphorylation in the oral epithelial cells of mice with OPC. (B) Representative histograms of $CD45^-$ $EpCam^+$ cells showing the effects of *C. albicans* infection and gefitinib (GEF) treatment on the phosphorylation of EGFR and EphA2 after 1 d of OPC. (C and D) Effects of gefitinib on the percentage of oral epithelial cells with phosphorylated EGFR (C) and EphA2 (D) in mice after 1 d of OPC. Data are combined results from 6 mice per group from a single experiment. Statistical significance was determined using the Mann-Whitney test. *, $p < 0.05$; **, $p < 0.01$; INF, infected; UNINF, uninfected. (E) Gating strategies used to identify $Ly6C^{hi}$ inflammatory monocytes and $Ly6C^+$ neutrophils in the flow cytometric analysis of the tongue digests. The results from these experiments are shown in Fig 6C and 6D.
