# Supplementary material for: Activation of EphA2-EGFR signaling in oral epithelial cells by Candida albicans virulence factors
Source: PLoS Pathog. 2021 Jan 20;17(1):e1009221. doi: 10.1371/journal.ppat.1009221 (PMC7850503; doi:10.1371/journal.ppat.1009221)
Supplement: S13 Fig — (A-C) The percentage of C. albicans cells killed by human neutrophils exposed to gefitinib ex vivo (A), bone marrow neutrophils isolated from gefitinib treated mice (B), and mouse bone marrow derived macrophages (BMDM) treated with gefitinib ex vivo (C). Data are the combined results of 3 experiments, each performed in duplicate. Data were analyzed using the two-tailed Student’s t-test assuming unequal variances. NS, not significant. (PDF) [file ppat.1009221.s013.pdf]

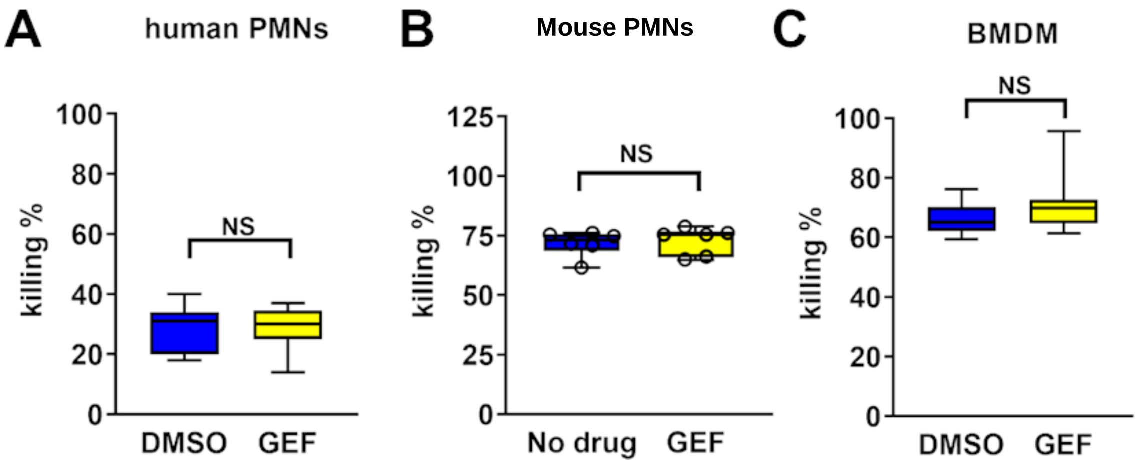

S13 Fig. Gefitinib has no effect on the killing of *C. albicans* by phagocytes. (A-C) The percentage of *C. albicans* cells killed by human neutrophils exposed to gefitinib *ex vivo* (A), bone marrow neutrophils isolated from gefitinib treated mice (B), and mouse bone marrow derived macrophages (BMDM) treated with gefitinib *ex vivo* (C). Data are the combined results of 3 experiments, each performed in duplicate. Data were analyzed using the two-tailed Student's t-test assuming unequal variances. NS, not significant.
