# Supplementary material for: Activation of EphA2-EGFR signaling in oral epithelial cells by Candida albicans virulence factors
Source: PLoS Pathog. 2021 Jan 20;17(1):e1009221. doi: 10.1371/journal.ppat.1009221 (PMC7850503; doi:10.1371/journal.ppat.1009221)
Supplement: S14 Fig — Oral fungal burden of immunocompetent C57BL/6 mice infected with indicated strains of C. albicans after 2 days of infection. Results are median of a total of 5 mice per group from a single experiment. The y-axis is set at the limit of detection (20 CFU/g tissue). Data were analyzed using the Mann-Whitney test.*, p < 0.05. (PDF) [file ppat.1009221.s014.pdf]

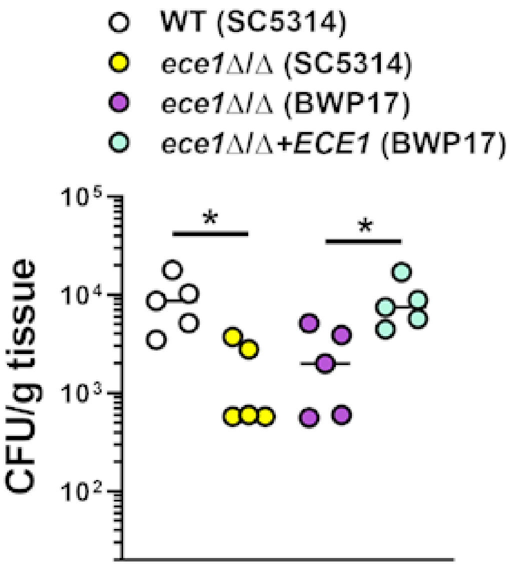

S14 Fig. Deletion of *ECE1* results in lower oral fungal burden. Oral fungal burden of immunocompetent C57BL/6 mice infected with indicated strains of *C. albicans* after 2 days of infection. Results are median of a total of 5 mice per group from a single experiment. The y-axis is set at the limit of detection (20 CFU/g tissue). Data were analyzed using the Mann-Whitney test.*, $p < 0.05$.
