# Supplementary material for: Activation of EphA2-EGFR signaling in oral epithelial cells by Candida albicans virulence factors
Source: PLoS Pathog. 2021 Jan 20;17(1):e1009221. doi: 10.1371/journal.ppat.1009221 (PMC7850503; doi:10.1371/journal.ppat.1009221)
Supplement: S15 Fig — Data are the combined results of three experiments, each performed in triplicate. Statistical significance was determined by the Brown-Forsythe and Welch ANOVA test. NS, not significant; orgs/HPF, organisms per high power field; WT, wild-type; *, p <0.05; p < 0.01; ***, p < 0.001; ****,p < 0.0001. (PDF) [file ppat.1009221.s015.pdf]

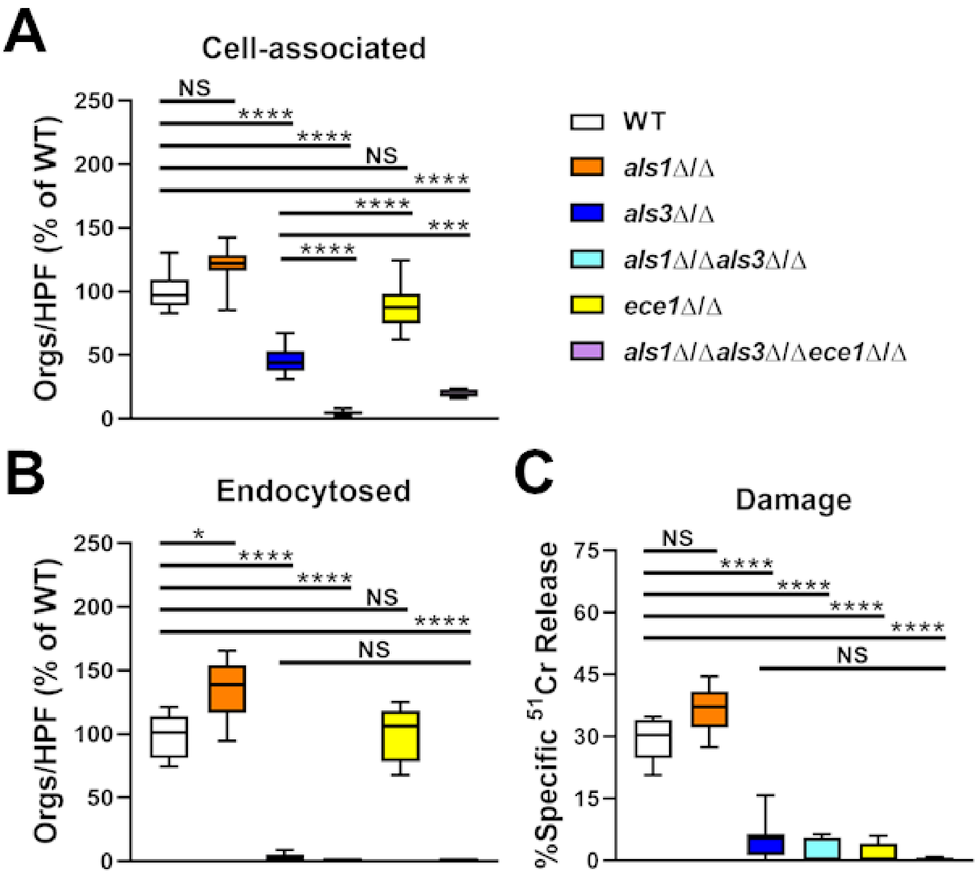

S15 Fig. Epithelial interactions of the indicated *C. albicans* strains. Data are the combined results of three experiments, each performed in triplicate. Statistical significance was determined by the Brown-Forsythe and Welch ANOVA test. NS, not significant; orgs/HPF, organisms per high power field; WT, wild-type; *, p <0.05; p < 0.01; ***, p < 0.001; ****,p < 0.0001.
