# Supplementary material for: Activation of EphA2-EGFR signaling in oral epithelial cells by Candida albicans virulence factors
Source: PLoS Pathog. 2021 Jan 20;17(1):e1009221. doi: 10.1371/journal.ppat.1009221 (PMC7850503; doi:10.1371/journal.ppat.1009221)
Supplement: S1 Table — (PDF) [file ppat.1009221.s016.pdf]

S1 Table. List of *C. albicans* strains used in the experiments.

Strain Number	Markers	Description / Genotype	Citation
SC5314		Wild-type	[1]
*ece1*Δ/Δ	His+<br>Arg+<br>Ura+	*ece1::HIS1/ece1::ARG4*;<br>*RPS1/rps1::URA3*	[2]
*ece1*Δ/Δ REV	His+<br>Arg+<br>Ura+	*ece1::HIS1/ece1::ARG4*;<br>*RPS1/rps1::URA3-ECE1*	[2]
MH216	NatR,<br>His-	*SC5314*<br>*his1Δ::r3NAT1r3/his1Δ::r3NAT1r3*	[3]
MH499	NatS,<br>His+	*his1Δ::r3/his1Δ::r3*<br>*ece1Δ::r1HIS1r1/ece1Δ::r1HIS1r1*	This study
MH562	NatS,<br>His+	*his1Δ::r3/his1Δ::r3*<br>*als3Δ::r1HIS1r1/als3Δ::r1HIS1r1*	This study
MH588	NatR,<br>His+	*his1Δ::r3/his1Δ::r3*<br>*als3Δ::r1HIS1r1/als3Δ::r1HIS1r1*<br>*ece1Δ::r3NAT1r3/ece1Δ::r3NAT1r3*	This study
JL018		*ece1Δ/ ece1Δ*	This study
JL030		*als1Δ/ als1Δ*	This study
JL036		*als3Δ/ als3Δ*	This study
JL050		*als1Δ/ als1Δ*<br>*als3Δ/ als3Δ*	This study
JL057		*ece1Δ/ ece1Δ*<br>*als1Δ/ als1Δ*<br>*als3Δ/ als3Δ*	This study
JL083		*ece1Δ/pECE1-ECE1-SAT1-tECE1*	This study
JL090		*ece1Δ/pECE1-ECE1-V5-SAT1-tECE1*	This study
JL092		*als3Δ/ als3Δ*<br>*ece1Δ/pECE1-ECE1-V5-SAT1-tECE1*	This study
JL095		*als3Δ/als3Δ*<br>*ece1Δ/pECE1-ECE1-SAT1-tECE1*	This study

References

1. Fonzi WA, Irwin MY. Isogenic strain construction and gene mapping in *Candida albicans*. Genetics. 1993;134(3):717-28.

2. Moyes DL, Wilson D, Richardson JP, Mogavero S, Tang SX, Wernecke J, et al. Candidalysin is a fungal peptide toxin critical for mucosal infection. Nature. 2016;532(7597):64-8.

3. Huang MY, Woolford CA, May G, McManus CJ, Mitchell AP. Circuit diversification in a biofilm regulatory network. PLoS Pathog. 2019;15(5):e1007787. Epub 2019/05/23. doi: 10.1371/journal.ppat.1007787. PubMed PMID: 31116789; PubMed Central PMCID: PMCPMC6530872.
