# Supplementary material for: Activation of EphA2-EGFR signaling in oral epithelial cells by Candida albicans virulence factors
Source: PLoS Pathog. 2021 Jan 20;17(1):e1009221. doi: 10.1371/journal.ppat.1009221 (PMC7850503; doi:10.1371/journal.ppat.1009221)
Supplement: S2 Table — (PDF) [file ppat.1009221.s017.pdf]

S2 Table. List of primers used in the experiments.

Name	Sequence (5’-3’)
sgRNA/F ECE1	tcatgttgaattctggagcaGTTTTAGAGCTAGAAATAGCAAGTTAAA
SNR52/R ECE1	tgctccagaattcaacatgaCAAATTAAAAATAGTTTACGCAAGTC
sgRNA/F ECE1-2	aatttctggcaatctgacgaGTTTTAGAGCTAGAAATAGCAAGTTAAA
SNR52/R ECE1-2	tcgtcagattgccagaaattCAAATTAAAAATAGTTTACGCAAGTC
sgRNA/F ALS3-5P	ATTGTTACTCATATATTTGTGTTTTAGAGCTAGAAATAGCAAGTTAAA
SNR52/R ALS3-5P	ACAAATATATGAGTAACAATCAAATTAAAAATAGTTTACGCAAGTC
sgRNA/F NAT1-5	GTCACGACGTTGTAAAACGAGTTTTAGAGCTAGAAATAGCAAGTTAAA
SNR52/R NAT1-5	TCGTTTTACAACGTCGTGACCAAATTAAAAATAGTTTACGCAAGTC
ECE1 check up/F	cacccaataggatcagtaaattctgc
ECE1 check int/R	atttggattacttgtggaatgttgc
ALS3 chk up/F	TATTGGCAACAACATCTTCCGC
ALS3 chk int/R	GAGTCAAAGTATTGCTCACAGTACATG
NAT1 Check/R	TCAATGGTGGATCAACTGGAACTTC
CdHIS1 Check Int/R	ggcgcaacagatatattggtgctcg
ALS3 del rHIS1rSapI/F	CCTCCCTTGAATTGAGGTCTGATAGTTTTTAATTTCATTTTATTATAATTGTATAAACAACTACCAACTGCTAATATTAGCTCGAGGTCGACGGTATCG
ALS3 del rHIS1rKpnI/R	AATTTTTTTTTTGGAGCCAAAAAAACAAAAAACAAACAAATAACAAAAATCTAAAAAGGCGACTATGATGGTATCATCCTCCAATACGCAAACCGCC
ECE1 del rHIS1rSapI/F	caaaattgttttatttttgtttatctctacaacaaacaactttcctttattttactaccaactattttccattcgttaaaCTCGAGGTCGACGGTATCG
ECE1 del rHIS1rKpnI/R	tcagttacagcaaaagtgtcacaagacttatggaataaaagattaagcttgtggaaaacaaatttttatctgctgagcatCCAATACGCAAACCGCC
ECE1 del rNATrBamHI/F	caaaattgttttatttttgtttatctctacaacaaacaactttcctttattttactaccaactattttccattcgttaaaGCTTTAATGCGGTAGTTTATCACAG
ECE1 del rNATrXMAI/R	tcagttacagcaaaagtgtcacaagacttatggaataaaagattaagcttgtggaaaacaaatttttatctgctgagcatGTGTGGTCGCCATGATCG
NAT1 CRIME/R	CACCATGACCTCTATGTTCTGG

NAT1 CRIME/F	CAGACGCGTTGAATTGTCC
HIS1 CRIME/F	gcgcaagaagcctcaact
HIS1 CRIME/R	gagctacagggcttgacc
Cas9-F	ATCTCATTAGATTTGGAACTTGTGGGTT
Cas9-R	TTCGAGCGTCCCAAAACCTTCT
SNR52_Far LF	AAGAAAGAAAGAAAACCAGGAGTGAA
SNF52	gcggccgcagtgattagact
ENO1_T_R	GCAGCTCAGTGATTAAGAGTAAAGATGG
ENO1_T_Far-R	ACAAATATTTAAACTCGGGACCTGG
gRNA_ScreenF	GGCTCGAACACAGTACCTCCAGA
gRNA_ScreenR	GGCGGCAAAACTAATTCTTCTCTT
CaCas9_ScreenF	AATTATCAAAAGACACCTATGACGACG
CaCas9_ScreenR	TCAACTGTTTCATCACTTTATCGTCAA
ALS1_Δ-F	CAATTGAAATGTGAAAGTTTGTTTTTTCGTTTTACTTCATCAGAATTGTTCAAACAACTACCAATTGTTAATATCAGggtaccgggccccccctcga
ALS1_Δ-R	TAATAATAACACGAAGAAAAGATAAATGTGAACTAGATCAAGCCAAAAAGGTGATCATAACAATATAGTCACCGCTCTAGAACTAGTGGATCTG
ALS1_gRNA_R	ATGGGGTTCTCCAGTAGTAAgttttagagctagaaatagcaagttaaa
ALS1_gRNA_F	TTACTACTGGAGAACCCCATcaaattaaaaatagtttacgcaagtc
ALS1_ORF F	ATGCTTCAACAATTTACATTG
ALS1_ORF R	TAGTTACGATTGAGGATTCATTGC
ALS3_Δ-F	CCCTTGAATTGAGGTCTGATAGTTTTTAATTTCATTTTATTATAATTGTATAAACAACTACCAACTGCTAATATTAGggtaccgggccccccctcga
ALS3_Δ-R	GGAGCCAAAAAAACAAAAAACAAACAAATAACAAAAATCTAAAAAGGCGACTATGATGGTATCATCCCCGCTCTAGAACTAGTGGATCTG
ALS3_gRNA_R	GTGCACCTTTCACATTAAGAgttttagagctagaaatagcaagttaaa
ALS3_gRNA_F	TCTTAATGTGAAAGGTGCACcaaattaaaaatagtttacgcaagtc
ALS3_ORF F	ATGCTACAACAATATACATTGTTAC
ALS3_ORF R	GGTAGTGTGATATGGAATATCAAC
ECE1_Δ-F	GTTTTATTTTTGTTTATCTCTACAACAAACAACTTTCCTTTATTTTACTACCAACTATTTTCCATTCGTTAAAggtaccgggccccccctcga
ECE1_Δ-R	CAGCAAAAGTGTCACAAGACTTATGGAATAAAAGATTAAGCTTGTGGAAAACAAATTTTTATCTGCTGAGCATTTCCGCTCTAGAACTAGTGGATCTG
ECE1_gRNA_R	ATTGTTGCTCGTGTTGCCACgttttagagctagaaatagcaagttaaa

ECE1_gRNA_F	GTGGCAACACGAGCAACAATcaaattaaaaatagtttacgcaagtc
ECE1_ORF F	ATGAAATTCTCCAAAATTGCC
ECE1_ORF R	GCAGATTCAGCTGATCTAG
ACT1-F	gtaaaacgacggccagtgaattcagcgtcaaaactagagaataat
Polylinker R	gaaacagctatgaccatgattacgccaagcttggcggccgctctagaactagtggatct
ECE1_promoterF	gtaaaacgacggccagtgaattcTCTTACATACAGGTGATATTGAG
ECE1_StopR	attctctagttttgacgctgaattcTTAAGCTTTTCCGAAATATTCTTC
ECE1_ter F	attactatttacaatcaaaggtggtcctgcagATGCTCAGCAGATAAAAATTTGTTTTC
ECE1_ter R	gaccatgattacgccaagcttggcggccgcTACTTAATTCAGTTGTTAGACAAGTTTTC
ECE1-C-V5-F	GGTAAGCCTATCCCTAACCCTCTCCTCGGTCTCGATTCTACGAACAAGAGAGAAGATATTGATTCTG
ECE1-C-V5-R	CGTAGAATCGAGACCGAGGAGAGGGTTAGGGATAGGCTTACCACCTTTGAAAGCTTTGACAATAC
