# Supplementary material for: Activation of EphA2-EGFR signaling in oral epithelial cells by Candida albicans virulence factors
Source: PLoS Pathog. 2021 Jan 20;17(1):e1009221. doi: 10.1371/journal.ppat.1009221 (PMC7850503; doi:10.1371/journal.ppat.1009221)
Supplement: S3 Table — (PDF) [file ppat.1009221.s018.pdf]

S3 Table. List of plasmids.

Plasmid Name	Description:	Bacterial Marker	Reference
pV1093	CaCas9/sgRNA expression vector	ampR	[1]
pSFS2A-mScarlet	pSFS2A carrying mScarlet, *MAL2* promoter driven flippase and *ACT1* promoter driven *NAT1*	chlR	[2]
pJL019	pUC19 backbone with T_{ACT1}-*SAT1* construct amplified from vector pSFS2A-mScarlet with primers ACT1-F/ PolylinkerR integrated at EcoRI/HindIII sites.	ampR	This Study
pJL034	pJL019 backbone with P_{ECE1}-*ECE1* fragment amplified from SC5314 genomic DNA with primers ECE1_promoterF/ECE1_StopR integrated at EcoRI site.	ampR	This Study
pJL044	pUC34 backbone with T_{ECE1} construct amplified with primers ECE1_terF/ECE1_terR integrated at PstI/NotI sites.	ampR	This Study
pJL081	pUC44 backbone with PECE1-ECE1-V5 construct integrated at EcoRI sites.	ampR	This Study
pMH05	YEp24 carrying *NAT1* from pNAT at BamHI site	ampR	[3]
pMH06	YEp24 carrying *NAT1* from pNAT at XmaI site	ampR	[3]
pMH01	pRS424 carrying *C.d.HIS1* from pSN52 at KpnI site	ampR	[4]
pMH02	pRS424 carrying *C.d.HIS1* from pSN52 at SapI site	ampR	[4]

References

1. Min K, Ichikawa Y, Woolford CA, Mitchell AP. *Candida albicans* gene deletion with a transient CRISPR-Cas9 system. MSphere. 2016;1(3):00130-16.

2. Frazer C, Hernday AD, Bennett RJ. Monitoring phenotypic switching in *Candida albicans* and the use of next-gen fluorescence reporters. Curr Protoc Microbiol. 2019;53(1):e76. Epub 2019/02/13. doi: 10.1002/cpmc.76. PubMed PMID: 30747494.

3. Huang MY, Woolford CA, May G, McManus CJ, Mitchell AP. Circuit diversification in a biofilm regulatory network. PLoS Pathog. 2019;15(5):e1007787. Epub 2019/05/23. doi: 10.1371/journal.ppat.1007787. PubMed PMID: 31116789; PubMed Central PMCID: PMCPMC6530872.

4. Huang MY, Mitchell AP. Marker Recycling in *Candida albicans* through CRISPR-Cas9-induced marker excision. mSphere. 2017;2(2). Epub 2017/03/21. doi: 10.1128/mSphere.00050-17. PubMed PMID: 28317025; PubMed Central PMCID: PMCPMC5352831.
